# Inhibition of bacterial biofilms by the snake venom proteome

**DOI:** 10.1016/j.btre.2023.e00810

**Published:** 2023-08-01

**Authors:** Neyaz A. Khan, Fernanda G. Amorim, John P. Dunbar, Dayle Leonard, Damien Redureau, Loïc Quinton, Michel M. Dugon, Aoife Boyd

**Affiliations:** a Pathogenic Mechanisms Research Group, School of Natural Sciences, University of Galway, Ireland; b Mass Spectrometry Laboratory, MolSys RU, University of Liège, Belgium; c Venom Systems & Proteomics Lab, School of Natural Sciences, Ryan Institute, University of Galway, Ireland

**Keywords:** Anti-biofilm agents, Snake venom, MRSA, MSSA, Anti-virulence molecules

## Abstract

Snake venoms possess a range of pharmacological and toxicological activities. Here we evaluated the antibacterial and anti-biofilm activity against methicillin-susceptible and methicillin-resistant *Staphylococcus aureus* (MSSA and MRSA) of venoms from the Samar spitting cobra *Naja samarensis* and the Puff adder *Bitis arietans.* Both venoms prevented biofilm production by pathogenic *S. aureus* in a growth-independent manner, with the *B. arietans* venom being most potent. Fractionation showed the active molecule to be heat-labile and >10 kDa in size. Proteomic profiles of *N. samarensis* venom revealed neurotoxins and cytotoxins, as well as an abundance of serine proteases and three-finger toxins, while serine proteases, metalloproteinases and C-lectin types were abundant in *B. arietans* venom. These enzymes may have evolved to prevent bacteria colonising the snake venom gland. From a biomedical biotechnology perspective, they have valuable potential for anti-virulence therapy to fight antibiotic resistant microbes.

## Highlights

• Snake venoms inhibit biofilm production by *Staphylococcus aureus*.
• *Naja samarensis* and *Bitis arietans* venom proteomics reveals abundance of proteases.
• Venom proteases could be developed for eradication of biofilm-producing bacteria.

## 1 Introduction

In 2019, 1.3 million deaths globally were directly linked to antimicrobial resistance (AMR) [1]. By the year 2050, these figures are expected to reach 10 million [2,3]. This is because of the rapid spread of AMR in the environment through excessive use of antibiotics [4], which are vectored into humans through insects and synanthropic animals [5], [6], [7], [8]. One key contributor to bacterial resistance is biofilm formation, where bacterial communities produce a complex protective layer of extracellular matrix that allows them to survive stressful conditions, evade antibiotics and host immune response [9]. Biofilm formation renders bacteria more antibiotic resistant compared to their planktonic form, making infections associated with biofilms difficult to treat [10]. Eighty percent of chronic infections are believed to be caused by biofilm [11]. Biofilm formation on prosthetic and implantable medical devices is associated with the majority of hospital acquired infections (HAIs) [12]. Among these, the majority of the infections are caused by *Staphylococcus aureus* [13]. A promising innovative approach to fighting biofilm-based infections is anti-virulence therapy which focuses on targeting virulence factors rather than directly killing the bacteria [14].

With over 220,000 species of venomous organisms, venoms are a rich source of pharmacologically active compounds with the ability to target specific biochemical pathways [15]. Animal venoms are composed of complex mixtures of biologically active toxic compounds that immobilize or kill prey by disrupting normal biological functions [16,17]. The bioactive molecules in venoms may also function in defence against colonisation of microbes and parasites within the venom gland [16,[18], [19], [20], [21]]. Despite this, some bacteria have adapted to survive in venom glands in complex microbial communities [22,23]. For example, a recent study recovered venom-resistant strains of *Enterococcus faecalis* from the venom of *Naja nigricollis. E. faecalis* is a clinically important pathogen linked to post-envenoming infections in Africa and Asia. Genomic analysis showed that these isolates contained up to 45 genes encoding proteins predicted to preserve bacterial membrane integrity and thereby to be involved in venom resistance [23].

For over a century, snake venoms have been exploited for developing life-saving anti-venoms. As a result, a great deal is known about the composition of several medically important snake venoms and translational applications of venom compounds as therapeutics is a successful and emerging field. The angiotensin-converting enzyme (ACE) inhibitors present in the venom of the South American pit viper *Bothrops jararaca* induce hypotension in victims of envenoming which subsequently led to the development of the FDA approved hypertensive drug Captopril [15]. More recently, snake venom phospholipase A2 has been shown to display virucidal activity against SARS‑CoV‑2 [24].

Previous investigations of snake venom components have identified constituent molecules that can interfere with the ability of bacteria to form biofilms. A study using viper venom reported phospholipase A2, an enzyme that hydrolyses phospholipids in biological membranes, as the active compound in the venom of *Bothrops erythromelas* responsible for anti-biofilm activity against *Acinetobacter baumanii* [21]. In support of the role of proteins and enzymes as anti-biofilm agents, three finger toxins (3FTx), L-amino acid oxidases and phospholipase A2 isolated from the cobra *Naja ashei* were present in the active fraction which inhibited *Staphylococcus epidermidis* biofilm formation [20].

We investigated the antimicrobial and anti-virulence activity of venoms from the African Puff adder *Bitis arietans* and Samar cobra *Naja samarensis*, a spitting species from the Philippines. Both of these species are listed by the World Health Organisation (WHO) as Category 1 medically important snakes [25]. A recent case series profiling the symptomology of envenoming reveals that the venom of *N. samarensis* can cause mild to extensive local cytotoxic to systemic neurotoxic envenoming [26]. We provide here proteomic profiles for the *B. arietans* and *N. samarensis* venoms investigated in this study and we demonstrate that the venoms of both *B. arietans* and *N. samarensis* possess anti-biofilm activity against methicillin susceptible *Staphylococcus aureus* (MSSA) and methicillin resistant *Staphylococcus aureus* (MRSA).

## 2 Material and methods

### 2.1 Bacterial strains and growth conditions

The bacterial strains used in this study were methicillin susceptible *Staphylococcus aureus* (MSSA) 8325-4 [27], methicillin resistant *Staphylococcus aureus* (MRSA) BH1CC [28], *Listeria monocytogenes* EGD-e [29], *Pseudomonas aeruginosa* PA01 [30] and *Vibrio parahaemolyticus* RIMD2210633 [31]. All bacterial strains were cultivated in brain heart infusion (BHI) (Oxoid) media, except *V. parahaemolyticus* (BHI + 3% NaCl), and grown at 37 °C.

### 2.2 Extraction of *N. samarensis* and *B. arietans* venoms

Venoms were pooled from two captive bred individual *B. arietans*. The *N. samarensis* venom sample was from a single captive bred individual. The snakes were maintained in a private collection in Ireland and raised on a strict diet of rats and mice. The snakes were made available to the authors for venom extraction, and samples were frozen at −20 °C then lyophilized before storage at 4 °C. The lyophilised venom was re-suspended in sterile water for testing.

### 2.3 Biofilm assay

The ability of snake venoms to inhibit biofilm formation of the target pathogens was assessed by crystal violet assay [32,33]. For biofilm assays, MSSA and MRSA cultures were grown in BHI supplemented with 1% glucose (w/v) and 4% NaCl (w/v), respectively. 200 μL overnight culture (adjusted to 0.02 OD_595_) was grown in the presence and absence of snake venoms in 2-fold dilutions from 3.75 to 0.02 mg/mL for *N. samarensis* and 0.34 to 0.005 mg/mL for *B. arietans* for 24 h at 37 °C. The experiment was carried out in Nunclon tissue culture-treated (delta surface) 96-well plates (Thermo Fisher). After 24 h the supernatant from each well was carefully removed and the biofilm was washed thrice with PBS. The biofilms were heat-fixed inside a 60 °C chamber for 1 h. 0.1% crystal violet (w/v) was added to stain the biofilms and incubated for 15 min at room temperature. The wells were washed thrice with PBS and images of the stained wells were obtained by photography. The stained biofilm was solubilised with 5% acetic acid (w/v) by mixing at room temperature for 15 min with agitation. Absorbance was measured at OD_595_. The amount of biofilm is directly proportional to the optical density value. Experiments were conducted thrice in triplicate.

### 2.4 Antibacterial activity of snake venoms

Antibacterial activity of *B. arietans* and *N. samarensis* venom was assessed by Minimum Inhibitory Concentration (MIC) assay. Overnight cultures of MRSA and MSSA were adjusted to 0.02 OD_595_ in BHI. Venoms were tested in triplicate against each pathogen in two-fold dilutions from 3.75 to 0.02 mg/mL for *N. samarensis* and 0.34 to 0.005 mg/mL for *B. arietans*. Gentamicin and vancomycin were used as positive controls. Samples were incubated for 24 h at 37 °C and either viewed directly or the absorbance at 600 nm was measured using a Tecan microplate reader with Magellan software. MIC is the minimum concentration of venom that results in complete inhibition of visual growth of bacteria.

The effect of *N. samarensis* venom on bacterial viability was assessed by time-kill assays against MRSA and MSSA [34]. 200 μL overnight bacterial culture (adjusted to 0.02 OD_595_) was incubated in the presence and absence of 3.75 mg/mL *N. samarensis* venom in BHI for 24 h at 37 °C. At 1, 2, 4 and 24 h time points, the cultures were serially diluted, 10 µL aliquots were spotted in triplicate onto BHI agar and incubated at 37 °C for 24 h. Colonies were counted and expressed as the number of colony forming units per mL (CFU/mL).

### 2.5 Preliminary characterisation of active component

100 μL venom (5 mg/mL) was loaded on Amicon Ultra 0.5 mL centrifugal filters (MWCO = 10 kDa) and centrifuged at 10,000 rpm for 30 min. The concentrate (>10 kDa) and the filtrate (<10 kDa) were collected and an aliquot (10 μL) of each was heat treated at 90 °C for 5 min. The heat-treated and untreated samples were then assessed for their anti-biofilm activity.

### 2.6 SDS-PAGE

Proteins were separated by SDS-PAGE and Coomassie stained according to standard protocols [35]. 20 µg sample of each venom was diluted in Laemlli buffer and heated for 3 min to 100 °C, before being separated alongside molecular weight markers using 1D SDS-PAGE NuPage (ThermoFisher Scientific) in MES SDS buffer. The resulting gel was first dehydrated with 50% (v/v) EtOH and 3% (v/v) phosphoric acid for 3 h, then rehydrated by means of a 20 min bath of ultrapure water (MilliQ). Coloration of the proteins was performed overnight with Coomassie blue (360 g/L, in an aqueous buffer with 34% (v/v) MeOH, 3% (v/v) phosphoric acid and 17% (w/v) ammonium sulphate). The gel was conserved at 5 °C in 5% (v/v) acetic acid.

### 2.7 Shotgun proteomics

10 µg of each lyophilized venom was dissolved in 20 µL 50 mM NH_4_HCO_3_ pH 7.8. The sample was reduced with 2 µL 500 mM dithiothreitol (DTT) for 40 min at 56 °C with shaking. The reduced samples were alkylated for 30 min at room temperature in the dark with 3 µL 500 mM iodoacetamide. After that, a second step of reduction was performed with 2 µL 500 mM DTT for 10 min at room temperature in the dark. Digestion with trypsin occurred in two consecutive steps: the first one at a ratio of 1:50 trypsin:protein with an overnight incubation at 37 °C and shaking at 650 rpm. The next day, a second step was performed with a trypsin:protein ratio of 1:100 at 37 °C for 3 h. The reactions were stopped by acidification with 10% (v/v, final concentration) Trifluoroacetic acid (TFA). Finally, the digested samples were dried by speed vacuum and prior to mass spectrometry analysis, the samples were suspended in 20 µL 0.1% (v/v) TFA for desalting on ZipTip pipette tips with C18 resin. Elution was performed with 20 µL 0.1% TFA/ACN (50/50, v/v).

The Liquid chromatography–tandem mass spectrometry (LC-MS/MS) analyses were performed on an Acquity M-Class UPLC (Waters) connected to a Q Exactive (Thermo Scientific) in nanoelectrospray positive ion mode. The trap column was a Symmetry C_18_ 5 μm (180 μm x 20 mm) and analytical column was a HSS T3 C_18_ 1.8 μm (75 μm x 250 mm) (Waters, Corp., Milford, USA). The samples were loaded at 20 μL/min on the trap column in 98% solvent A over 3 min and subsequently separated on the analytical column at a flow rate of 600 nL/min with the following linear gradient: initial conditions 98% A; 5 min 93% A; 60 min 70% A; 70 min 60% A, 73 min 15% A, maintained for 5 min, then the column was reconditioned to initial conditions. Solvent A was 0.1% formic acid in water and solvent B was 0.1% formic acid in acetonitrile. The total run time was 100 min. The mass spectrometer method was a TopN-MSMS method where N was set to 12, meaning that the spectrometer acquires one Full MS spectrum, selects the 12 most intense peaks in this spectrum (singly charged and unassigned charge precursors excluded) and makes a Full MS2 spectrum of each of these 12 compounds. The parameters for MS spectrum acquisition were Mass range from 400 to 1750 m/z; Resolution of 70,000; AGC target of 1e6 or maximum injection time of 200 ms. The parameters for MS2 spectrum acquisition were: isolation window of 2.0 m/z; Normalized Collision Energy (NCE) of 25; Resolution of 17,500; AGC target of 1e5 or maximum injection time of 50 ms. The main parameters for Q Exactive tune were spray voltage of 2.3 kV, capillary temperature of 270 °C and S-Lens RF level of 50.0.

Protein identification by automated de novo sequencing was performed using the software Peaks Studio *X*+ [36], with database created by the deposits related to “Snake” and “Venom” family in the UniProt repository, downloaded in November 2021 (74,759 sequences) (Figure S1 and S2). Carbamidomethylation was set as fixed modification and oxidation (M) were set as variable modification, with maximum missed cleavages at 3. Parent mass and fragment mass error tolerances were set at 5 ppm and 0.015 Da, respectively. A false discovery rate (FDR) of 0.1% and unique peptide ≥ 1 were used to filter out inaccurate proteins for the PEAKS search algorithms and “De novo only” analysis with *a* − 10lgP > 20 for the database match with high in confidence. The top proteins were used for venom component classification. The relative percentage of the proteins in each digested venom was estimated as described by Zainal Abidin and colleagues [37], using the following formula: [number of proteins (protein family) / total number of proteins detected using LC-MSMS] x 100 (Figure S3 and S4).

### 2.8 Statistical analysis

The data were presented as mean ± standard deviation (SD). The P-values were calculated using Student's *t*-test. *P* < 0.05 was considered as significant. IC_50_ of the venoms were calculated by applying appropriate polynomial regression model to describe the relationship between percent biofilm inhibition and venom concentration.

## 3 Results

### 3.1 Anti-biofilm activity of *B. arietans* and *N. samarensis* venoms

**Fig. 1:**
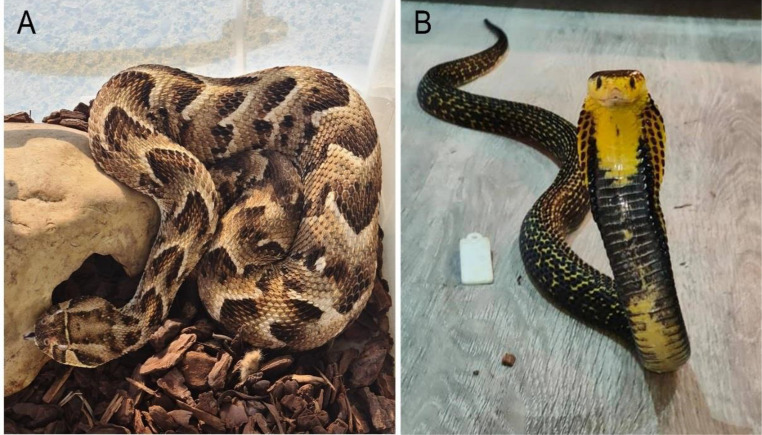
The venoms used in this study were extracted from A) African viper - Puff adder *Bitis arietans* and B) Samar cobra *Naja samarensis.* (photo credit: Mark Mosley).

Venoms of 2 medically important snakes - *N. samarensis* (Elapidae family) and *B. arietans* (Viperidae family) (Fig. 1) - were investigated for their anti-biofilm activity against MRSA, MSSA, *L. monocytogenes, P. aeruginosa* and *V. parahaemolyticus*. These target bacterial pathogens cause severe infections in human, such as sepsis, bacteraemia and gastroenteritis [38], [39], [40], [41]. Little information is known about the antimicrobial and anti-biofilm properties of these snake venoms, whereas venoms of other snakes within these families showed a broad range of antimicrobial activity against both Gram-positive and Gram-negative bacteria [42].

**Fig. 2:**
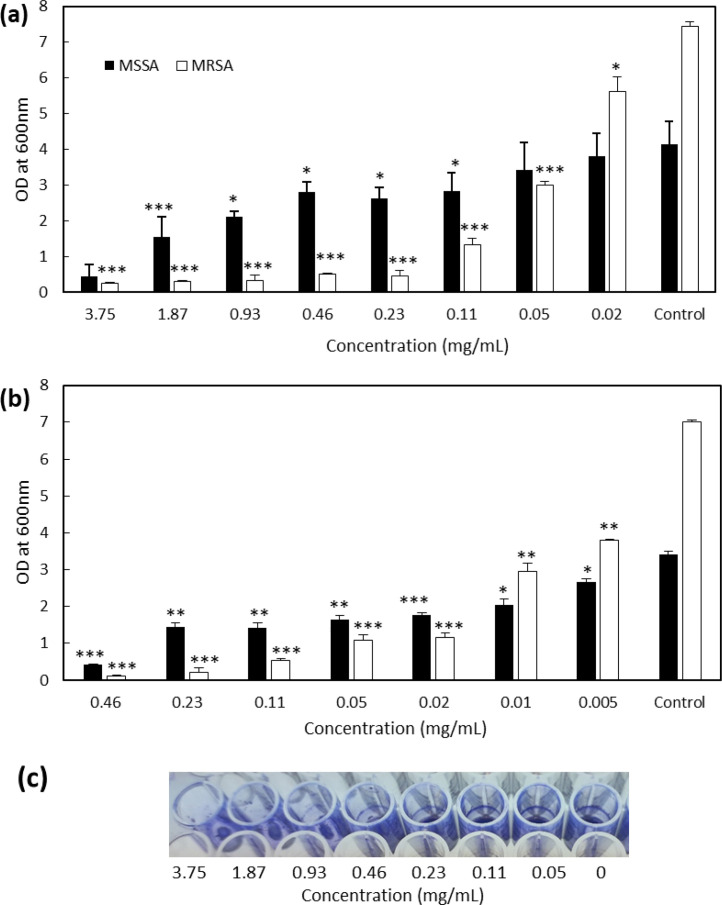
Inhibition of MRSA and MSSA biofilm production by venoms of *N. samarensis* and *B. arietans*. Bacteria were cultured with and without the venom of *N. samarensis* (a) and *B. arietans* (b). Biofilm formed after 24 h incubation at 37 °C was stained with crystal violet and after solubilisation, OD_600_ was measured. Prior to solubilisation an image of crystal violet-stained MRSA biofilm was obtained (c). Data presented are the average ±SD of three independent experiments performed in triplicate. P-values were calculated by comparing the samples with corresponding control (* = *p <* 0.05, ** = *p <* 0.01, *** = *p <* 0.001).

The biofilm inhibition assay was performed by culturing the bacteria in microtitre 96 well-plates in the presence of serial dilutions of the venoms for 24 h.  After incubation, the amount of attached biofilm was measured by staining with crystal violet. Venoms were analysed at the maximum concentration possible based on the amount of venom available, meaning that they were assessed at varied concentration ranges: 0.02–3.75 mg/mL for *N. samarensis* venom and 0.005–0.46 mg/mL for *B. arietans* venom. Neither of the venoms inhibited biofilm formation of *L. monocytogenes, P. aeruginosa* or *V. parahaemolyticus* (data not shown). However, both venoms showed anti-biofilm activity against *S. aureus* by completely inhibiting the biofilm formation of MSSA and MRSA (Fig. 2a and b) at the highest concentrations tested.

Venom of *N. samarensis* inhibited MSSA biofilm formation by 90% at 3.75 mg/mL (Fig. 2a). At 1.87 and 0.93 mg/mL venom biofilm inhibition was 63 and 50%, respectively. Similar biofilm inhibition of 31–36% was observed for 0.46, 0.23 and 0.11 mg/mL venom. There was no significant inhibition for lower concentrations. Venom of *N. samarensis* showed stronger biofilm inhibition activity against MRSA than MSSA with 95% biofilm inhibition at 0.23 mg/mL (Fig. 2a). There was dose-dependent biofilm inhibition with 82, 60 and 25% biofilm inhibition at 0.11, 0.05 and 0.02 mg/mL venom, respectively. The IC_50_ of *N. samarensis* venom was 0.80 ± 0.27 and 0.04 ± 0.003 mg/mL towards MSSA and MRSA, respectively.

On the other hand, venom of *B. arietans* showed 88% reduction in MSSA biofilm production at 0.46 mg/mL (Fig. 2b). This effect is almost 8 times stronger than the anti-biofilm activity of *N. samarensis* against MSSA (Fig. 2a). MSSA biofilm inhibition of 50–60% was observed for *B. arietans* venom at concentrations ranging from 0.23 to 0.02 mg/mL (Fig. 2b). Dose-dependent inhibition was observed with 42 and 22% inhibition at 0.01 and 0.005 mg/mL venom, respectively. Similar to anti-biofilm activity of *N. samarensis* venom (Fig. 2a), the venom of *B. arietans* showed stronger anti-biofilm activity against MRSA than MSSA, with more than 90% reduction in biofilm at 0.08 mg/mL (Fig. 2b). This effect is 3 times stronger than effect of *N. samarensis* venom against MRSA (Fig. 2a). Dose dependent biofilm inhibition was observed with 85%, 60 and 46% inhibition at 0.02, 0.01 and 0.005 mg/mL venom, respectively. The IC_50_ of *B. arietans* venom was 0.093 ± 0.025 and 0.0068 ± 0.0002 mg/mL towards MSSA and MRSA, respectively.

**Fig. 3:**
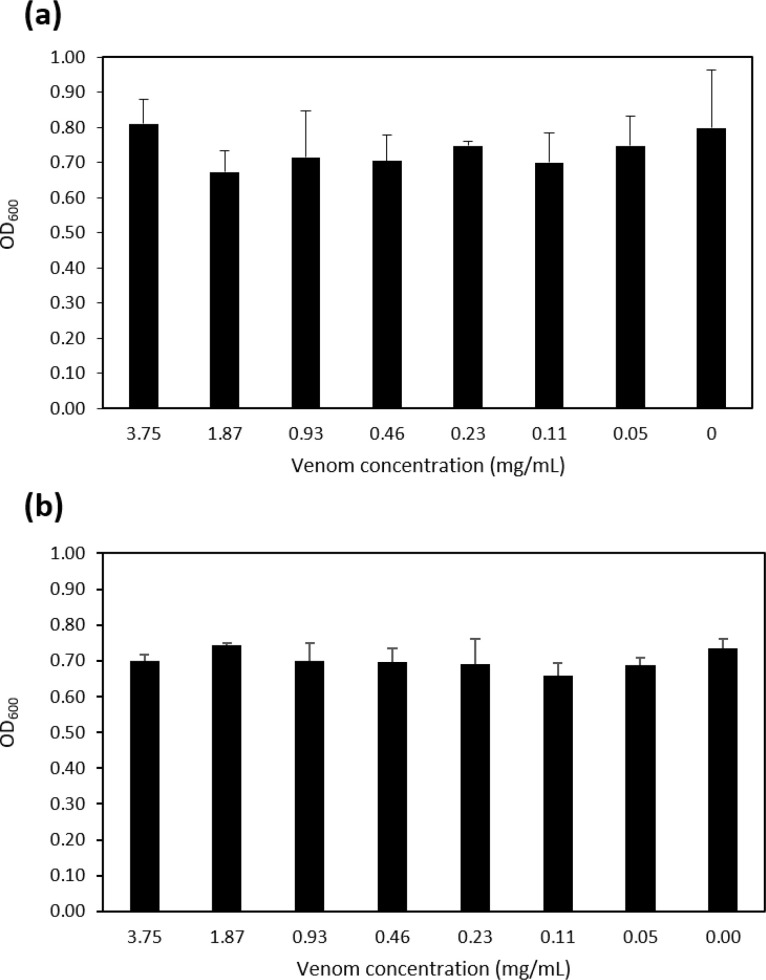
*N. samarensis* venom does not reduce growth of *S. aureus*. Venom at the indicated concentrations was incubated with (a) MRSA or (b) MSSA in BHI at 37 °C for 24 h and the OD_600_ measured. Data are the average ± SD of three biological replicates assessed in triplicate.

**Fig. 4:**
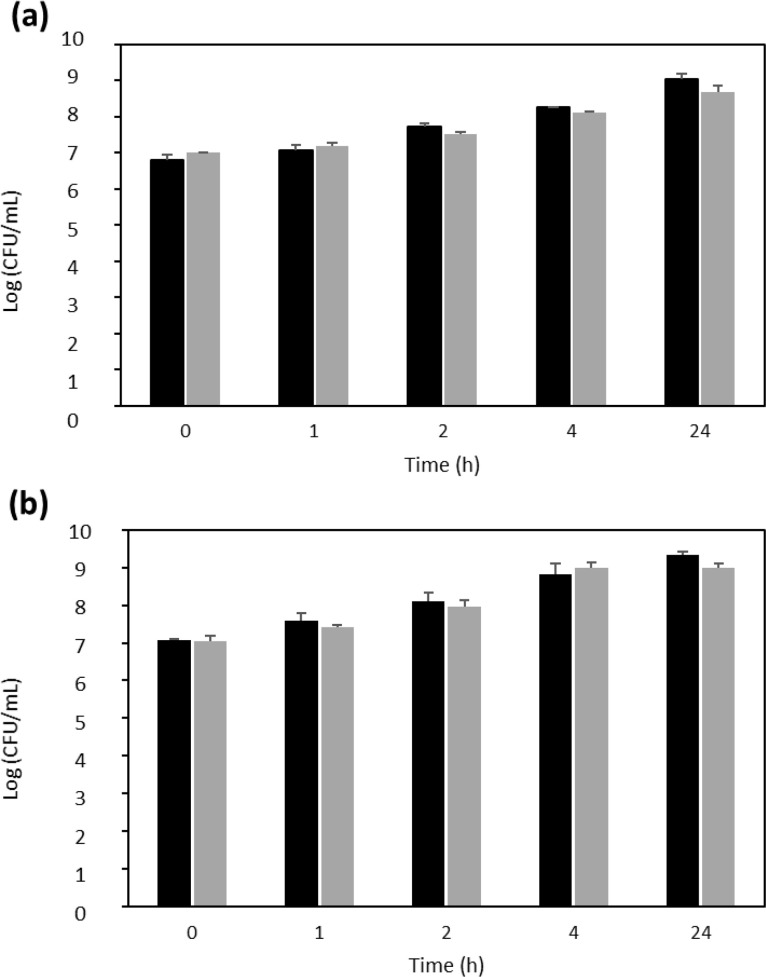
*N. samarensis* venom does not reduce viability of *S. aureus*. 3.75 mg/mL venom was incubated with 0.02 OD (a) MRSA or (b) MSSA in BHI at 37 °C for the indicated times. Aliquots were then serially diluted and spotted in triplicate on BHI plates to determine the CFU. Data are the average ± SD of three biological replicates. Black columns indicate untreated control bacteria and grey columns indicate bacteria incubated with venom.

Anti-virulence molecules should target specifically the virulence factors of the bacteria and not negatively affect bacterial growth. We therefore investigated the effect of the venoms on the growth of MRSA and MSSA. After 24 h, there was no visible difference in the growth of the bacteria and the absorbance at OD_600_ for each pathogen in the presence of the highest concentration of venom was 0.27 ± 0.01 (MSSA) and 0.35 ± 0.03 (MRSA) for *N. samarensis* and 0.31 ± 0.02 (MSSA) and 0.39 ± 0.04 (MRSA) for *B. arietans* which was very similar to growth in the absence of venom (0.28 ± 0.01 and 0.37 ± 0.06, respectively). We further examined the antimicrobial activity of *N. samarensis* venom through MIC and viability assays. There was no effect of the venom at any concentration on growth of either *S. aureus* strain after 24 h (Fig. 3). Similarly, 3.75 mg/mL venom did not reduce the viability of MRSA or MSSA at any time point (Fig. 4). This indicates that neither venom had antibacterial activity against *S. aureus* and that their ability to inhibit biofilm formation is independent of growth inhibition.

In conclusion, venoms of *N. samarensis* and *B. arietans* possess anti-biofilm activity against both MSSA and MRSA with *B. arietans* having higher activity. Both venoms have stronger anti-biofilm activity against MRSA than MSSA.

### 3.2 Preliminary characterisation of the active venom component

**Fig. 5:**
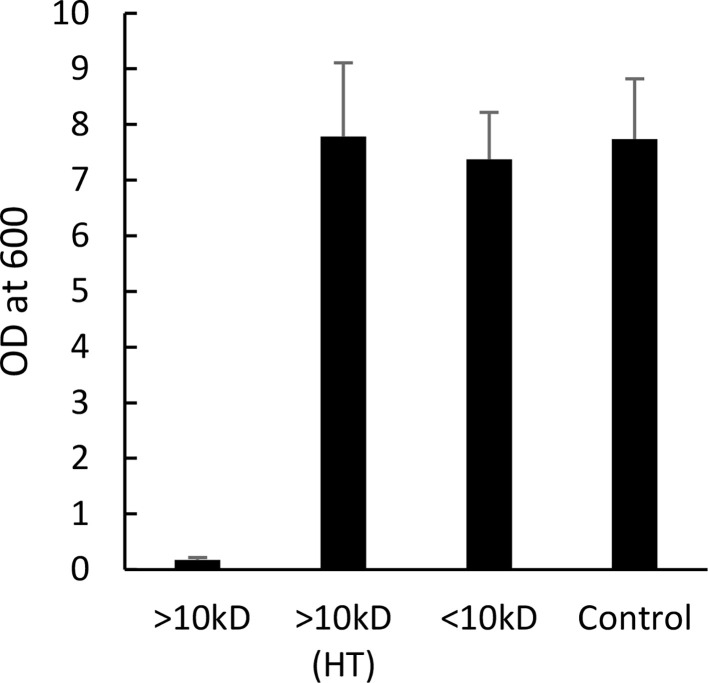
MRSA biofilm production in the presence of *N. samarensis* venom fractions. *N. samarensis* venom was fractionated into <10 kDa and >10 kDa molecules. Heat-treated (HT) and untreated fractions were tested for anti-biofilm activity against MRSA. Data presented are the average ± SD of two independent experiments performed in triplicate.

To determine the size and nature of the active component, we fractionated the *N. samarensis* venom by size (<10 kDa and >10 kDa) using ultrafiltration. This venom was chosen due to the amount of venom available at that time. The fraction with >10 kDa molecules showed strong anti-biofilm activity by completely inhibiting MRSA biofilm production compared to the control, whereas no activity was detected for the <10 kDa fraction (Fig. 5). Moreover, upon heat treatment the >10 kDa fraction lost its anti-biofilm activity. This suggested the anti-biofilm molecule(s) could be proteinaceous.

### 3.3 SDS-PAGE analysis of *N. samarensis* and *B. arietans* venoms

**Fig. 6:**
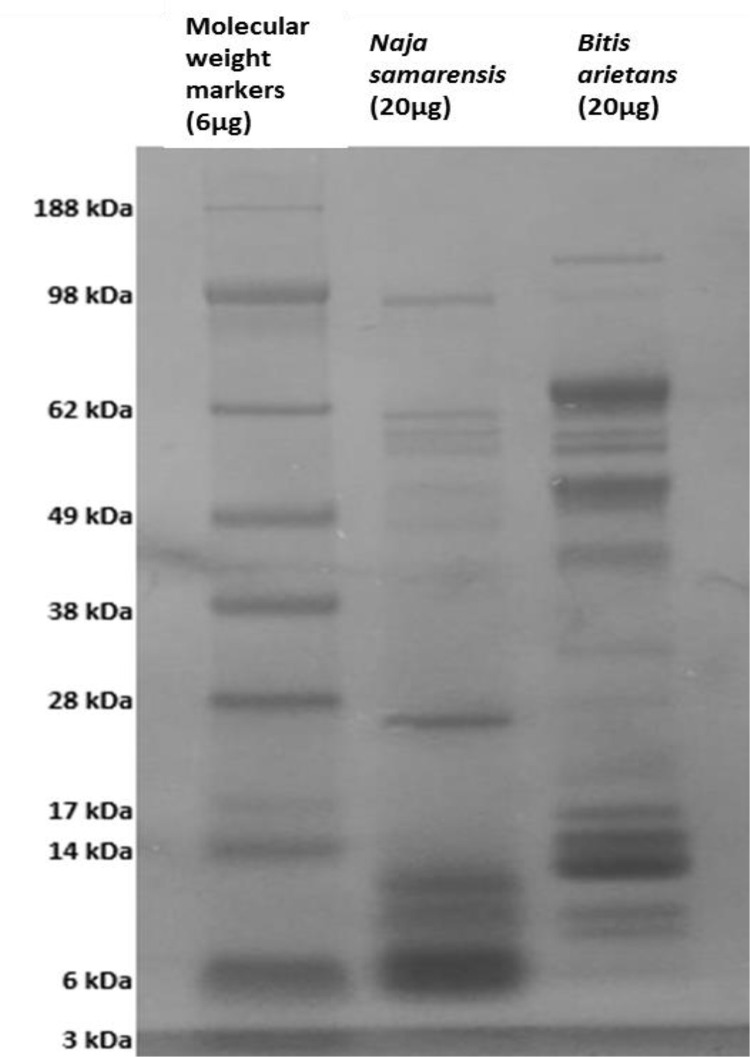
1D SDS-PAGE analysis of crude venoms from *N. samarensis* and *B. arietans*. 20 µg sample of each venom was separated using 1D SDS-PAGE, followed by Coomassie blue staining.

SDS-PAGE analysis was performed to generate the protein profile of each venom (Fig. 6). The electrophoretic separation and Coomassie staining of each venom sample revealed a range of intense bands. Venom obtained from *N. samarensis* displayed a group of proteins between 6 and 12 kDa and a ∼26-kDa protein was clearly visible. Also visible in lower abundance were proteins ranging from 45 to 100 kDa. Venom from *B. arietans* had a quite different protein profile. It possessed abundant proteins ranging from 12 to 17 kDa and from 40 to 70 kDa. Fewer proteins between 6 and 12 kDa were present than in *N. samarensis* venom, while on the other hand a prominent protein of around 130 kDa was detected. In order to produce a detailed overview of the molecular composition of these venoms, a relative quantitative proteomics approach was undertaken.

### 3.4 Proteomic analysis of *N. samarensis* and *B. arietans* venom

Venom profiles of *B. arietans* and *N. samarensis* were generated using a shotgun approach. The proteins identified in the proteomes are presented in the Supplementary Material (Table S1, S2, S3 and S4). Less than 10% of the total proteome (4.8–7.8%) could not be classified as they did not show similarity with existing protein families and additionally a proportion were classified as cellular components (35.5% in *N. samarensis* and 17% in *B. arietans*) (Table 1). The venom proteins were divided into two groups: characterised envenoming toxins – molecules that have been reported to be toxic to human cells and/or tissues in other venomous species - and additional venom proteins – proteins with as yet unknown roles in envenoming.

**Table 1:** Proteomic study showing relative distribution of protein families in *N. samarensis* and *B. arietans* venoms.

Protein family	*N. samarensis* (%)	*B. arietans* (%)
**Characterised Envenoming Toxins**		
3FTx	15.2	1.8
Snake venom serine proteinase	13.8	21.2
Snake venom metalloproteinase	7.4	14.5
Phospholipase A2	2.8	4.2
Hyaluronidase	1.8	1.2
Venom Kunitz-type family	0.5	3.0
L-amino acid oxidase	0.5	0
C-lectin type	0	12.7
Disintegrin	0	3.6
**Additional Venom Proteins**		
Venom complement C3-like	2.8	0
Cysteine-rich venom protein	2.3	6.1
Venom endothelial growth factor	1.8	0.6
5′-nucleotidase family	1.4	3.6
Phospholipase inhibitor	1.4	0
Cathepsin	1.4	0
Aminopeptidase	0.9	1.8
Phospholipase B	0.9	0.6
Phosphodiesterase	0.5	1.8
Cystatin	0.5	0.6
Nerve growth factor	0.5	0.6
Vespryn	0.5	0
**Cellular and Unassigned Proteins**		
Cellular component	35.5	17.0
Protein family not assigned	7.8	4.8

Through analysis of relative distribution and abundance of the venom protein families (Table 1), it can be observed that there are significant differences in proteome diversity of the two snake venoms, as may be expected from two such distantly related genera. In the venom of *N. samarensis* there are proteins of 19 different venom protein families: 3FTx (15.2%), snake venom serine proteinases (13.8%) and snake venom metalloproteinases (7.4%) are the most abundant. In the venom of *Bitis arietans*, there are 16 different protein families, among which snake venom serine proteinases (21.2%), snake venom metalloproteinases (14.5%), C-lectin types (12.7%) and cysteine-rich venom proteins (6.1%) are the most abundant. The *N. samarensis* and *B. arietans* venoms share 14 venom protein families with some variation in relative abundance. For example, snake venom serine proteinases are much more abundant in *B. arietans* compared to *N. samarensis,* whereas both venoms have low amounts of cystatins, nerve growth factor and phospholipases B.

## 4 Discussion

The emergence of AMR bacteria poses a serious threat to human health [43]. Bacteria have developed resistance to almost all frontline antibiotics and developing new antibiotics is one of the topmost priorities to combat these resistant pathogens. Antimicrobials with novel mechanisms are required to minimise bacterial resistance development and this can be achieved by exploring novel sources of antimicrobial compounds which can target virulence factors of the pathogens in a growth-independent manner. In this context exploration of snake venoms is an emerging field with promising potential for discovering new antimicrobials.

Here, we demonstrate the ability of venom from *B. arietans* and *N. samarensis* to inhibit biofilm formation by MSSA and MRSA in a growth-independent manner by quantitative and qualitative analysis of crystal violet-stained biofilm material (Fig. 2). We suggest that the role of the anti-biofilm molecules is not necessarily to prevent disease and infection, but rather to maintain a venom gland that is unobstructed by the build-up of biofilm of commensal and/or environmental bacteria of the microflora community so that venom can freely flow during envenoming. The active component(s) of the venom were shown to be >10-kDa heat-labile molecule(s) (Fig. 5). This excluded peptides and other small molecules as the active compound and instead pointed towards a proteinaceous anti-biofilm agent.

The venoms only showed anti-biofilm activity against *S. aureus* and not against the other pathogens tested. We speculate that this may be explained by the specific degradation by venom proteases of S*. aureus* adhesin proteins utilised for attachment to surfaces [44]. Proteases were the most abundant proteins in both venoms. While many bacterial species possess adhesin proteins [45], it is possible that the venom proteases specifically degrade *S. aureus* adhesin proteins (for example, FnBP). Of the two venoms, *B. arietans* showed the strongest activity against MSSA and MRSA. This correlated with the higher abundance of proteases (43.6%) in the venom proteome of *B. arietans* compared to *N. samarensis* (24.4%) (Table 1). We hypothesise that proteases are amongst the active anti-biofilm molecules in these venoms.

In general, compounds may inhibit biofilm formation either directly as AVM targeting biofilm production pathways or indirectly through their antimicrobial activity. An example of the indirect approach are recent studies reporting anti-biofilm activity of snake venoms where 3FTx (<10 kDa), L-amino acid oxidases (50–70 kDa) and phospholipase A2 (13–15 kDa) protein families have emerged as key components inhibiting biofilm formation [20,21]. Several studies have demonstrated the antibacterial activity of these proteins against both Gram-negative and Gram-positive bacteria, including *S. aureus* [46], [47], [48]. Therefore, the prevention of bacterial growth is likely the cause of the decreased biofilm production associated with these proteins, rather than direct anti-virulence activity per se. Proteins belonging to these three families were present in both the *B. arietans* and *N. samarensis* venoms. It may therefore be unexpected that no antibacterial activity was detected in our study. We showed by MIC and time-kill assays that the venom did not prevent or reduce growth of *S. aureus* nor decrease its survival and viability (Fig. 3 and 4). This could be due to structural variations in the proteins and/or due to differential protein abundance between the snake species.

We focussed on confirming the lack of antibacterial activity of *N. samarensis* venom as its antibacterial potential has, to the best of our knowledge, not been previously investigated. In conditions of the biofilm development assay with 3.75 mg/mL *N. samarensis* venom no adverse effects were detected on bacterial growth, as assessed by broth dilution MIC assays, or on survival, as assessed by CFU bacterial viability assays (Figs. 3 and 4). Previous investigations on the potential antibacterial activity of *B. arietans* venom utilised either agar or disk diffusion assays. Blaylock *et al*. demonstrated anti-*S. aureus* activity of neat non-quantified venom [49]. Al-Asmari et al. concluded that only at concentrations > 1 mg/mL (total amount 50 µg) did *B. arietans* venom display antimicrobial activity against *S. aureus* and that 0.5 mg/mL venom (25 µg) had no effect [50]. The work of Okumu et al. also showed that 25 µg venom did not affect *S. aureus* growth [51]. Our results indicate that at 460 µg/mL the venom of *B. arietans* does not reduce growth of *S. aureus* in broth assays.

Regarding the direct anti-biofilm AVM approach, only a couple of studies have previously demonstrated anti-biofilm activity of snake venom that is independent of effects on bacterial growth. Both studies reported anti-biofilm activity against *S. aureus* of lectin extracted from venom of *Bothrops jararacussu* [19,52]. Our proteomic analysis revealed the presence of C-lectin type proteins in *B. arietans* which may suggest a correlation to the stronger activity of *B. arietans* compared to *N. samarensis*.

The venom proteome is critical during envenoming in the host. Each year snakebite mortality exceeds 150,000 deaths [53]. More than 30,000 deaths are recorded annually in sub-Saharan Africa [54,55], though this figure is thought to be considerably underestimated due to potentially unreliable national data [53,54]. *B. arietans* is believed to be one of the species responsible for the majority of these deaths [56]. 13,377 snakebites resulting in 550 deaths are predicted to occur annually in the Philippines [57]. *N. samarensis* is endemic to the Visayas and Mindanao Island groups of the archipelago and is listed as a category 1 species of medical importance. Whilst little is known about the symptomology of envenoming of *N. samarensis*, a recent report suggests that victims experience typical systemic neurotoxicity and mild to extensive local cytotoxicity (i.e. necrosis) [26]. Our proteomic analysis provides a catalogue of the proteins that may account for these symptoms.

There have been previous endeavours to explore the venom profiles of *B. arietans* [58], [59], [60], [61], [62], [63], [64]. Our data shares both similarities and differences with previous proteomic profiling of *B. arietans* venom [58,60,63,64]. In three studies serine proteases, metalloproteinases and C-lectin types were amongst the most abundant venom protein families, similar to our data [58,63,64]. For example, the work by Dingwoke et al. [64] revealed proportions of snake venom serine proteinase and C-lectin type (22.3% and 10.7% of total venom, respectively) comparable to those obtained to the current study (21.2% and 12.7%, respectively). Their study also revealed similar, though slightly higher, percentages of snake venom metalloproteinases (21.1% vs 14.5%), phospholipases A2 (10.6% vs 4.2%), and similar, though slightly lower, proportions of cysteine-rich secretory proteins (2.1% vs 6.1%) and Kunitz-type family proteins (1.1% vs 3.0%). The venom profile characterized by Wang et al. was quite different with disintegrin, C-lectin type and 3FTx being the most highly represented protein families, whereas serine protease and metalloproteinase together accounted for less than 10% [60]. Disintegrins were also present at a high percentage (17.8%) in the study of Juarez et al. [58], but at a low percentage in the other 3 proteomes, while 3FTx were either not identified or were at a low percentage in the other 4 proteomes. Most of the additional protein families ranged from 0 to 5% relative percentage in each proteome.

Our data for 3FTx in the *N. samarensis* venom proteome contrasts sharply with the results obtained by Palasuberniam et al. [65] which revealed a high content of 3FTx (90.5% of total venom) compared to the 15.2% 3FTx detected in this study. There were similar relative percentages of snake venom metalloproteinases (4.2% vs 7.4%), phospholipase A2 (3.8% vs 2.8%), cysteine-rich secretory protein (1.1% vs 2.3%), L-amino acid oxidase (0.3% vs 0.5%), venom nerve growth factor (0.1% vs 0.5%) and vespryn (0.1% vs 0.5%) in the two studies. Their study also revealed snake venom serine proteinase was absent (0%), whereas in our sample, snake venom serine proteinase constituted 13.8% of total venom. Serine proteinase is usually more common in vipers than in cobras [66] and can contribute to coagulopathies [67] and cause shock from hypotension in envenomed victims [68].

The reasons for the differences between venom proteomes of different individuals, or groups of individuals, belonging to the same snake species are not clear and this topic has been receiving recent attention [69,70]. The differences in venom composition can result in highly variable snakebite pathologies and affect anti-venom efficacy [71]. The hunting and feeding ecology of snakes likely drives inter- and intra-species variation in venom composition. Geographical location has been proposed as a cause of variation, as well as the environment of the specimens prior to venom extraction [70]. The *N. samarensis* venom analysed by Palasuberniam et al. was pooled from five wild-caught specimens from the southern Philippines [65], and the *B. arietans* venom examined by Dingwoke et al. was pooled from six wild-caught male adult specimens from regions north and south of Nigeria [64]. In comparison, our *N. samarensis* venom sample was obtained from a single captive-bred specimen, and our *B. arietans* venom sample was taken from two captive-bred specimens in a private collection in Ireland. This reinforces the potential importance of animal origin and management prior to venom extraction, which may account for significant venom variations and in turn, have an impact on envenoming management.

In conclusion, this work demonstrates that *B. arietans* and *N. samarensis* venom exhibit strong anti-biofilm activity against both MRSA and MSSA and have anti-virulence activities. Proteomic studies highlighted several protein families that could be responsible for this activity. We propose that the anti-biofilm activity of the *B. arietans* is a combination of lectin and protease activity, while that of *N. samarensis* depends more on proteases. Future work would focus on isolating and characterising these anti-biofilm compounds. This study indicates that venoms are ideal candidates in the search for novel anti-virulence molecules in the fight against AMR bacterial infections.

## Ethical statement

In accordance with Directive 2010/63/EU a project authorisation by the Health Products Regulatory Authority (HPRA) under scientific animal protection legislation was not required to conduct this study.

## CRediT authorship contribution statement

**Neyaz A. Khan:** Conceptualization, Data curation, Formal analysis, Funding acquisition, Investigation, Methodology, Software, Validation, Visualization, Writing – original draft, Writing – review & editing. **Fernanda G. Amorim:** Data curation, Formal analysis, Funding acquisition, Investigation, Methodology, Software, Validation, Writing – review & editing. **John P. Dunbar:** Conceptualization, Funding acquisition, Methodology, Writing – review & editing. **Dayle Leonard:** Formal analysis, Investigation, Writing – review & editing. **Damien Redureau:** Investigation, Methodology, Writing – review & editing. **Loïc Quinton:** Funding acquisition, Methodology, Resources, Supervision, Writing – review & editing. **Michel M. Dugon:** Conceptualization, Funding acquisition, Methodology, Resources, Supervision, Writing – review & editing. **Aoife Boyd:** Conceptualization, Data curation, Funding acquisition, Methodology, Project administration, Resources, Supervision, Validation, Visualization, Writing – review & editing.

## Declaration of Competing Interest

The authors declare that they have no known competing financial interests or personal relationships that could have appeared to influence the work reported in this paper.

## Data Availability

Data will be made available on request.

## Appendix Supplementary materials

Image, application 1

Image, application 2

Image, application 3

Image, application 4
