# Supplementary material for: Inhibition of bacterial biofilms by the snake venom proteome
Source: Biotechnol Rep (Amst). 2023 Aug 1;39:e00810. doi: 10.1016/j.btre.2023.e00810 (PMC10407894; doi:10.1016/j.btre.2023.e00810)
Supplement: Supplementary file 1 [file mmc1.docx]

Table S2. Proteomic data and protein family identification of *Naja samarensis* venom

| **Protein Group** | **Protein ID** | **Accession** | **-10lgP** | **Coverage (%)** | **Coverage (%) Sample 1** | **Area Sample 1** | **#Peptides** | **#Unique** | **#Spec Sample 1** | **Post Translation Modification** | **Average Mass** | **Description** | **Protein Family** |
| --- | --- | --- | --- | --- | --- | --- | --- | --- | --- | --- | --- | --- | --- |
| 3 | 1 | A0A2D0TC04\|PDE_NAJAT | 324.91 | 33 | 33 | 1.76E+09 | 38 | 38 | 50 | Carbamidomethylation | 94616 | Venom phosphodiesterase OS=Naja atra OX=8656 PE=1 SV=1 | Phosphodiesterases |
| 13 | 15 | Q9PVK7\|VM3_NAJKA | 285.97 | 24 | 24 | 6.36E+08 | 21 | 5 | 28 | Carbamidomethylation; Oxidation (M) | 67662 | Zinc metalloproteinase-disintegrin-like cobrin OS=Naja kaouthia OX=8649 PE=2 SV=1 | Snake venom metalloproteinases |
| 5 | 86 | Q92084\|PA2NA_NAJSP | 282.02 | 62 | 62 | 1.86E+08 | 24 | 3 | 43 | Carbamidomethylation | 16189 | Neutral phospholipase A2 muscarinic inhibitor OS=Naja sputatrix OX=33626 PE=1 SV=1 | Phospholipases A2 |
| 10 | 2 | A0A2I4HXH5\|V5NTD_NAJAT | 280.99 | 40 | 40 | 6.44E+08 | 29 | 9 | 38 | Carbamidomethylation; Oxidation (M) | 58198 | Snake venom 5'-nucleotidase (Fragment) OS=Naja atra OX=8656 PE=1 SV=1 | 5’-nucleotidase family |
| 15 | 6 | I2C090\|VCO3_OPHHA | 274.58 | 12 | 12 | 4.00E+08 | 23 | 14 | 32 | Carbamidomethylation | 183927 | Ophiophagus venom factor OS=Ophiophagus hannah OX=8665 PE=1 SV=1 | Venom complement C3-likes |
| 26 | 204 | Q5YF89\|NGFV2_NAJSP | 262.77 | 34 | 34 | 1.82E+09 | 12 | 12 | 20 | Carbamidomethylation; Oxidation (M) | 27030 | Venom nerve growth factor 2 OS=Naja sputatrix OX=33626 PE=2 SV=1 | Nerve growth factors |
| 20 | 3 | tr\|A0A194AS98\|A0A194AS98_9SAUR | 257.26 | 30 | 30 | 1.93E+06 | 21 | 1 | 27 | Carbamidomethylation | 63012 | Ecto-5'-nucleotidase OS=Micrurus tener OX=1114301 PE=3 SV=1 | 5’-nucleotidase family |
| 20 | 4 | tr\|U3FYP9\|U3FYP9_MICFL | 257.26 | 30 | 30 | 1.93E+06 | 21 | 1 | 27 | Carbamidomethylation | 62983 | Ecto-5'-nucleotidase OS=Micrurus fulvius OX=8637 PE=2 SV=1 | 5’-nucleotidase family |
| 22 | 30 | D3TTC2\|VM3H_NAJAT | 256.63 | 18 | 18 | 7.75E+06 | 15 | 1 | 22 | Carbamidomethylation; Oxidation (M) | 69181 | Zinc metalloproteinase-disintegrin-like atragin OS=Naja atra OX=8656 PE=1 SV=1 | Snake venom metalloproteinases |
| 17 | 336 | tr\|A0A0U5ARS4\|A0A0U5ARS4_NAJNA | 241.42 | 51 | 51 | 2.36E+08 | 14 | 6 | 29 | Carbamidomethylation; Oxidation (M) | 7959 | Cytotoxin 11 (Fragment) OS=Naja naja OX=35670 GN=CTX11 PE=2 SV=1 | Venom complement C3-likes |
| 14 | 16 | A8QL58\|OXLA_NAJAT | 240.24 | 29 | 29 | 6.58E+08 | 18 | 18 | 33 | Carbamidomethylation; Oxidation (M) | 57963 | L-amino-acid oxidase (Fragment) OS=Naja atra OX=8656 PE=1 SV=2 | L-amino-acid oxidases |
| 8 | 218 | A4FS04\|PA2A_NAJAT | 233.43 | 58 | 58 | 3.55E+06 | 13 | 1 | 40 | Carbamidomethylation; Oxidation (M) | 13188 | Acidic phospholipase A2 natratoxin OS=Naja atra OX=8656 PE=1 SV=2 | Phospholipases A2 |
| 18 | 99 | P00596\|PA2A1_NAJKA | 231.17 | 51 | 51 | 3.83E+05 | 16 | 2 | 26 | Carbamidomethylation | 16271 | Acidic phospholipase A2 CM-II OS=Naja kaouthia OX=8649 PE=1 SV=2 | Phospholipases A2 |
| 23 | 819 | Q9PSN6\|3S13_NAJSP | 229.5 | 74 | 74 | 8.41E+07 | 12 | 1 | 25 | Carbamidomethylation | 6958 | Neurotoxin 3 OS=Naja sputatrix OX=33626 PE=1 SV=1 | 3-finger toxins |
| 23 | 825 | tr\|B0FXL8\|B0FXL8_9SAUR | 229.5 | 55 | 55 | 8.41E+07 | 12 | 1 | 25 | Carbamidomethylation | 9263 | Siamenotoxin I (Fragment) OS=Naja siamensis OX=84476 GN=VW PE=2 SV=1 | 3-finger toxins |
| 19 | 822 | P60771\|3S1CB_NAJKA | 229.35 | 57 | 57 | 3.18E+09 | 14 | 3 | 29 | Carbamidomethylation | 9262 | Cobrotoxin OS=Naja kaouthia OX=8649 PE=1 SV=2 | 3-finger toxins |
| 19 | 824 | Q9PTT0\|3S1CB_NAJNA | 229.35 | 57 | 57 | 3.18E+09 | 14 | 3 | 29 | Carbamidomethylation | 9262 | Cobrotoxin homolog OS=Naja naja OX=35670 PE=1 SV=1 | 3-finger toxins |
| 19 | 823 | P60770\|3S1CB_NAJAT | 229.35 | 57 | 57 | 3.18E+09 | 14 | 3 | 29 | Carbamidomethylation | 9262 | Cobrotoxin OS=Naja atra OX=8656 PE=1 SV=1 | 3-finger toxins |
| 7 | 168 | P01445\|3SA7A_NAJKA | 226.4 | 98 | 98 | 0 | 15 | 1 | 29 | Carbamidomethylation; Oxidation (M) | 6745 | Cytotoxin 2 OS=Naja kaouthia OX=8649 PE=1 SV=1 | 3-finger toxins |
| 7 | 169 | tr\|A0A0U4N5W4\|A0A0U4N5W4_NAJNA | 226.4 | 82 | 82 | 0 | 15 | 1 | 29 | Carbamidomethylation; Oxidation (M) | 7947 | Cytotoxin 13 (Fragment) OS=Naja naja OX=35670 GN=CTX13 PE=2 SV=1 | 3-finger toxins |
| 21 | 56 | P84805\|CRVP1_NAJKA | 223.27 | 45 | 45 | 1.19E+09 | 17 | 13 | 24 | Carbamidomethylation | 26846 | Cysteine-rich venom protein kaouthin-1 OS=Naja kaouthia OX=8649 PE=1 SV=2 | Cysteine-rich venom proteins |
| 30 | 112 | P84808\|CRVP2_NAJKA | 216.77 | 29 | 29 | 2.69E+08 | 8 | 8 | 13 | Carbamidomethylation | 26216 | Cysteine-rich venom protein kaouthin-2 OS=Naja kaouthia OX=8649 PE=1 SV=2 | Cysteine-rich venom proteins |
| 30 | 113 | tr\|A0A024AX20\|A0A024AX20_MICIK | 216.77 | 29 | 29 | 2.69E+08 | 8 | 8 | 13 | Carbamidomethylation | 26216 | Cysteine-rich secretory protein OS=Micropechis ikaheca OX=66188 PE=2 SV=1 | Cysteine-rich venom proteins |
| 24 | 354 | P14541\|3SOFH_NAJKA | 203.06 | 77 | 77 | 4.01E+09 | 10 | 10 | 22 | Carbamidomethylation | 6994 | Cytotoxin homolog OS=Naja kaouthia OX=8649 PE=1 SV=1 | 3-finger toxins |
| 24 | 355 | P62377\|3SOFL_NAJNA | 203.06 | 77 | 77 | 4.01E+09 | 10 | 10 | 22 | Carbamidomethylation | 7014 | Cytotoxin-like basic protein OS=Naja naja OX=35670 PE=1 SV=1 | 3-finger toxins |
| 24 | 356 | P0DUK7\|3SOF1_NAJAT | 203.06 | 77 | 77 | 4.01E+09 | 10 | 10 | 22 | Carbamidomethylation | 7062 | Mu-elapitoxin-Na1a OS=Naja atra OX=8656 PE=1 SV=1 | 3-finger toxins |
| 24 | 397 | P62375\|3SOF5_NAJAT | 203.06 | 58 | 58 | 4.01E+09 | 10 | 10 | 22 | Carbamidomethylation | 9323 | Cytotoxin A5 OS=Naja atra OX=8656 PE=1 SV=1 | 3-finger toxins |
| 24 | 398 | Q91996\|3SOFP_NAJAT | 203.06 | 58 | 58 | 4.01E+09 | 10 | 10 | 22 | Carbamidomethylation | 9305 | Cardiotoxin 7'' OS=Naja atra OX=8656 PE=3 SV=1 | 3-finger toxins |
| 24 | 608 | Q9W716\|3SOFV_NAJAT | 203.06 | 58 | 58 | 4.01E+09 | 10 | 10 | 22 | Carbamidomethylation | 9333 | Cytotoxin homolog 5V OS=Naja atra OX=8656 PE=3 SV=1 | 3-finger toxins |
| 11 | 317 | P60308\|3SAFC_NAJAT | 201.58 | 70 | 70 | 2.89E+05 | 9 | 1 | 11 | Carbamidomethylation | 6755 | Cytotoxin SP15c OS=Naja atra OX=8656 PE=1 SV=1 | 3-finger toxins |
| 33 | 92 | tr\|A0A670Z5C6\|A0A670Z5C6_PSETE | 192.78 | 17 | 17 | 8.81E+07 | 8 | 2 | 11 | Carbamidomethylation | 50029 | T-plasminogen activator OS=Pseudonaja textilis OX=8673 GN=PLAT PE=3 SV=1 | Snake venom serine proteinases |
| 31 | 77 | Q0ZZJ6\|VCO31_AUSSU | 187.78 | 4 | 4 | 6.48E+07 | 7 | 1 | 11 | Carbamidomethylation | 184725 | A.superbus venom factor 1 OS=Austrelaps superbus OX=29156 PE=1 SV=1 | Venom complement C3-likes |
| 31 | 78 | tr\|Q49HM6\|Q49HM6_AUSSU | 187.78 | 4 | 4 | 6.48E+07 | 7 | 1 | 11 | Carbamidomethylation | 184893 | Venom factor OS=Austrelaps superbus OX=29156 PE=2 SV=1 | Venom complement C3-likes |
| 39 | 181 | D5LMJ3\|VM3A_NAJAT | 178.34 | 8 | 8 | 1.37E+08 | 6 | 6 | 7 | Carbamidomethylation | 68254 | Zinc metalloproteinase-disintegrin-like atrase-A OS=Naja atra OX=8656 PE=2 SV=1 | Snake venom serine proteinases |
| 43 | 300 | tr\|A0A6I9YJI0\|A0A6I9YJI0_9SAUR | 173.85 | 12 | 12 | 9.00E+06 | 6 | 1 | 9 | Carbamidomethylation | 50565 | T-plasminogen activator OS=Thamnophis sirtalis OX=35019 GN=PLAT PE=3 SV=1 | Snake venom serine proteinases |
| 34 | 175 | tr\|A0A081DUA7\|A0A081DUA7_ECHCO | 169.59 | 3 | 3 | 1.35E+06 | 7 | 1 | 10 | Carbamidomethylation | 185008 | Complement C3a OS=Echis coloratus OX=64175 PE=2 SV=1 | Venom complement C3-likes |
| 32 | 811 | P82463\|3SUC2_NAJKA | 164.23 | 63 | 63 | 2.78E+09 | 5 | 4 | 10 | Carbamidomethylation | 7298 | Muscarinic toxin-like protein 2 OS=Naja kaouthia OX=8649 PE=1 SV=1 | 3-finger toxins |
| 41 | 378 | A8QL53\|VSP1_NAJAT | 163.29 | 11 | 11 | 1.79E+08 | 5 | 5 | 7 | Carbamidomethylation | 31137 | Snake venom serine protease NaSP (Fragment) OS=Naja atra OX=8656 PE=2 SV=1 | Snake venom serine proteinases |
| 41 | 385 | A8QL57\|VSP1_BUNMU | 163.29 | 11 | 11 | 1.79E+08 | 5 | 5 | 7 | Carbamidomethylation | 31010 | Snake venom serine protease BmSP (Fragment) OS=Bungarus multicinctus OX=8616 PE=2 SV=1 | Snake venom serine proteinases |
| 41 | 504 | tr\|A0A670ZDU7\|A0A670ZDU7_PSETE | 163.29 | 11 | 11 | 1.79E+08 | 5 | 5 | 7 | Carbamidomethylation | 29915 | Peptidase S1 domain-containing protein OS=Pseudonaja textilis OX=8673 PE=3 SV=1 | Snake venom serine proteinases |
| 41 | 505 | tr\|A0A670ZGC3\|A0A670ZGC3_PSETE | 163.29 | 11 | 11 | 1.79E+08 | 5 | 5 | 7 | Carbamidomethylation | 29881 | Peptidase S1 domain-containing protein OS=Pseudonaja textilis OX=8673 PE=3 SV=1 | Snake venom serine proteinases |
| 41 | 506 | tr\|A0A670ZGB3\|A0A670ZGB3_PSETE | 163.29 | 11 | 11 | 1.79E+08 | 5 | 5 | 7 | Carbamidomethylation | 30015 | Peptidase S1 domain-containing protein OS=Pseudonaja textilis OX=8673 PE=3 SV=1 | Snake venom serine proteinases |
| 41 | 698 | tr\|A0A6J1TU04\|A0A6J1TU04_9SAUR | 163.29 | 12 | 12 | 1.79E+08 | 5 | 5 | 7 | Carbamidomethylation | 28056 | serine protease harobin isoform X3 OS=Notechis scutatus OX=8663 GN=LOC113411373 PE=3 SV=1 | Snake venom serine proteinases |
| 41 | 699 | tr\|A0A6J1TUS8\|A0A6J1TUS8_9SAUR | 163.29 | 11 | 11 | 1.79E+08 | 5 | 5 | 7 | Carbamidomethylation | 28988 | serine protease harobin isoform X2 OS=Notechis scutatus OX=8663 GN=LOC113411373 PE=3 SV=1 | Snake venom serine proteinases |
| 41 | 718 | tr\|A0A6J1TVU9\|A0A6J1TVU9_9SAUR | 163.29 | 10 | 10 | 1.79E+08 | 5 | 5 | 7 | Carbamidomethylation | 31530 | snake venom serine protease NaSP isoform X1 OS=Notechis scutatus OX=8663 GN=LOC113411373 PE=3 SV=1 | Snake venom serine proteinases |
| 40 | 1182 | Q9YGI4\|3NO22_NAJAT | 160.19 | 27 | 27 | 4.07E+07 | 5 | 1 | 9 | Carbamidomethylation; Oxidation (M) | 9899 | Probable weak neurotoxin NNAM2 OS=Naja atra OX=8656 PE=1 SV=1 | 3-finger toxins |
| 40 | 1345 | O42256\|3NO26_NAJSP | 160.19 | 27 | 27 | 4.07E+07 | 5 | 1 | 9 | Carbamidomethylation; Oxidation (M) | 9807 | Weak neurotoxin 6 OS=Naja sputatrix OX=33626 PE=3 SV=1 | 3-finger toxins |
| 40 | 1346 | Q802B3\|3NO28_NAJSP | 160.19 | 27 | 27 | 4.07E+07 | 5 | 1 | 9 | Carbamidomethylation; Oxidation (M) | 9809 | Weak neurotoxin 8 OS=Naja sputatrix OX=33626 PE=3 SV=2 | 3-finger toxins |
| 40 | 1347 | O42255\|3NO25_NAJSP | 160.19 | 27 | 27 | 4.07E+07 | 5 | 1 | 9 | Carbamidomethylation; Oxidation (M) | 9806 | Weak neurotoxin 5 OS=Naja sputatrix OX=33626 PE=3 SV=1 | 3-finger toxins |
| 40 | 1344 | P60814\|3NO2I_NAJAT | 160.19 | 27 | 27 | 4.07E+07 | 5 | 1 | 9 | Carbamidomethylation; Oxidation (M) | 9811 | Probable weak neurotoxin NNAM2I OS=Naja atra OX=8656 PE=3 SV=1 | 3-finger toxins |
| 29 | 202 | tr\|V8P395\|V8P395_OPHHA | 159.58 | 25 | 25 | 4.74E+08 | 5 | 5 | 10 | Carbamidomethylation | 29588 | Glutathione peroxidase (Fragment) OS=Ophiophagus hannah OX=8665 GN=Gpx3 PE=3 SV=1 | Cellular components |
| 53 | 233 | tr\|A0A0B8RWS3\|A0A0B8RWS3_BOIIR | 158.77 | 22 | 22 | 6.51E+06 | 4 | 4 | 5 |  | 22790 | Peptidyl-prolyl cis-trans isomerase OS=Boiga irregularis OX=92519 PE=3 SV=1 | Cellular components |
| 53 | 234 | tr\|U3FWC8\|U3FWC8_MICFL | 158.77 | 22 | 22 | 6.51E+06 | 4 | 4 | 5 |  | 22829 | Peptidyl-prolyl cis-trans isomerase OS=Micrurus fulvius OX=8637 PE=2 SV=1 | Cellular components |
| 53 | 237 | tr\|U3F781\|U3F781_MICFL | 158.77 | 21 | 21 | 6.51E+06 | 4 | 4 | 5 |  | 24656 | Peptidyl-prolyl cis-trans isomerase OS=Micrurus fulvius OX=8637 PE=2 SV=1 | Cellular components |
| 25 | 593 | P01452\|3SA4_NAJMO | 155.25 | 52 | 52 | 1.27E+08 | 6 | 1 | 8 | Carbamidomethylation | 6715 | Cytotoxin 4 OS=Naja mossambica OX=8644 PE=1 SV=1 | 3-finger toxins |
| 38 | 394 | P60774\|3S11_NAJSA | 150.3 | 75 | 75 | 1.62E+08 | 6 | 5 | 10 | Carbamidomethylation | 6818 | Short neurotoxin 1 OS=Naja samarensis OX=8660 PE=1 SV=1 | 3-finger toxins |
| 38 | 395 | P60772\|3S15_NAJSP | 150.3 | 75 | 75 | 1.62E+08 | 6 | 5 | 10 | Carbamidomethylation | 6818 | Neurotoxin 5 OS=Naja sputatrix OX=33626 PE=1 SV=1 | 3-finger toxins |
| 35 | 842 | Q9YGJ6\|3S1A1_NAJSP | 145.38 | 55 | 55 | 1.05E+08 | 7 | 2 | 11 | Carbamidomethylation | 9220 | Alpha-neurotoxin NTX-1 OS=Naja sputatrix OX=33626 PE=3 SV=1 | 3-finger toxins |
| 35 | 843 | O57326\|3S1A3_NAJSP | 145.38 | 55 | 55 | 1.05E+08 | 7 | 2 | 11 | Carbamidomethylation | 9289 | Alpha-neurotoxin NTX-3 OS=Naja sputatrix OX=33626 PE=3 SV=1 | 3-finger toxins |
| 50 | 657 | tr\|V8NYC7\|V8NYC7_OPHHA | 144.8 | 12 | 12 | 7.80E+07 | 3 | 1 | 5 | Carbamidomethylation | 31729 | Tissue-type plasminogen activator (Fragment) OS=Ophiophagus hannah OX=8665 GN=PLAT PE=3 SV=1 | Snake venom serine proteinases |
| 55 | 292 | tr\|V8P1Y2\|V8P1Y2_OPHHA | 144.64 | 6 | 6 | 1.01E+07 | 4 | 4 | 4 |  | 83039 | Neuroendocrine convertase 1 (Fragment) OS=Ophiophagus hannah OX=8665 GN=PCSK1 PE=3 SV=1 | Snake venom serine proteinases |
| 45 | 829 | P82464\|3SO8_NAJKA | 142.54 | 46 | 46 | 3.17E+07 | 5 | 2 | 7 | Carbamidomethylation | 7624 | Muscarinic toxin-like protein 3 OS=Naja kaouthia OX=8649 PE=1 SV=1 | 3-finger toxins |
| 58 | 1275 | P82942\|VM3K_NAJKA | 141.09 | 9 | 9 | 4.39E+07 | 3 | 1 | 3 | Carbamidomethylation | 44493 | Hemorrhagic metalloproteinase-disintegrin-like kaouthiagin OS=Naja kaouthia OX=8649 PE=1 SV=1 | Snake venom metalloproteinases |
| 42 | 101 | tr\|A0A081DUC1\|A0A081DUC1_PANGU | 140.01 | 4 | 4 | 8.01E+07 | 6 | 1 | 7 | Carbamidomethylation | 185574 | Complement C3 OS=Pantherophis guttatus OX=94885 PE=2 SV=1 | Venom complement C3-likes |
| 52 | 275 | tr\|V8NQ76\|V8NQ76_OPHHA | 136.36 | 7 | 7 | 2.83E+06 | 4 | 4 | 4 |  | 77681 | Atriopeptidase OS=Ophiophagus hannah OX=8665 GN=MME PE=3 SV=1 | Snake venom serine proteinases |
| 70 | 619 | E3P6P4\|CYT_NAJKA | 132.45 | 18 | 18 | 1.42E+07 | 3 | 3 | 3 |  | 15772 | Cystatin OS=Naja kaouthia OX=8649 PE=2 SV=1 | Cystatins |
| 46 | 826 | P82849\|3S1B2_NAJKA | 127.08 | 58 | 58 | 1.25E+08 | 4 | 2 | 7 | Carbamidomethylation | 6862 | Cobrotoxin II OS=Naja kaouthia OX=8649 PE=1 SV=1 | 3-finger toxins |
| 63 | 859 | tr\|V8N4Y2\|V8N4Y2_OPHHA | 126.56 | 25 | 25 | 2.74E+07 | 3 | 3 | 3 | Carbamidomethylation | 18083 | Endonuclease domain-containing 1 protein OS=Ophiophagus hannah OX=8665 GN=ENDOD1 PE=4 SV=1 | Cellular components |
| 61 | 511 | tr\|A6MJH7\|A6MJH7_PSEAU | 125.79 | 4 | 4 | 3.22E+06 | 3 | 3 | 3 | Carbamidomethylation | 86495 | Dipeptidyl peptidase 4 OS=Pseudechis australis OX=8670 PE=2 SV=1 | Cellular components |
| 61 | 512 | tr\|A6MJH6\|A6MJH6_CRYNI | 125.79 | 4 | 4 | 3.22E+06 | 3 | 3 | 3 | Carbamidomethylation | 86617 | Dipeptidyl peptidase 4 OS=Cryptophis nigrescens OX=292442 PE=2 SV=1 | Cellular components |
| 61 | 579 | tr\|A0A077LAF7\|A0A077LAF7_PROFL | 125.79 | 4 | 4 | 3.22E+06 | 3 | 3 | 3 | Carbamidomethylation | 86179 | Venom dipeptidylpeptidase IV OS=Protobothrops flavoviridis OX=88087 PE=2 SV=1 | Cellular components |
| 61 | 513 | tr\|A6MJI0\|A6MJI0_HOPST | 125.79 | 4 | 4 | 3.22E+06 | 3 | 3 | 3 | Carbamidomethylation | 86405 | Dipeptidyl peptidase 4 OS=Hoplocephalus stephensii OX=196418 PE=2 SV=1 | Cellular components |
| 61 | 577 | tr\|U3TDD4\|U3TDD4_PROFL | 125.79 | 4 | 4 | 3.22E+06 | 3 | 3 | 3 | Carbamidomethylation | 79450 | Dipeptidyl peptidase iv OS=Protobothrops flavoviridis OX=88087 PE=2 SV=1 | Cellular components |
| 61 | 547 | tr\|A6MJI1\|A6MJI1_TROCA | 125.79 | 4 | 4 | 3.22E+06 | 3 | 3 | 3 | Carbamidomethylation | 86457 | Dipeptidyl peptidase 4 OS=Tropidechis carinatus OX=100989 PE=2 SV=1 | Cellular components |
| 61 | 520 | tr\|A6MJH4\|A6MJH4_PSETE | 125.79 | 4 | 4 | 3.22E+06 | 3 | 3 | 3 | Carbamidomethylation | 86559 | Dipeptidyl peptidase 4 OS=Pseudonaja textilis OX=8673 PE=2 SV=1 | Cellular components |
| 61 | 517 | tr\|A6MJH8\|A6MJH8_PSEPO | 125.79 | 4 | 4 | 3.22E+06 | 3 | 3 | 3 | Carbamidomethylation | 86607 | Dipeptidyl peptidase 4 OS=Pseudechis porphyriacus OX=8671 PE=2 SV=1 | Cellular components |
| 61 | 544 | tr\|A6MJH5\|A6MJH5_DEMVE | 125.79 | 4 | 4 | 3.22E+06 | 3 | 3 | 3 | Carbamidomethylation | 85960 | Dipeptidyl peptidase 4 OS=Demansia vestigiata OX=412038 PE=2 SV=1 | Cellular components |
| 61 | 545 | tr\|A6MJH2\|A6MJH2_OXYSU | 125.79 | 4 | 4 | 3.22E+06 | 3 | 3 | 3 | Carbamidomethylation | 86600 | Dipeptidyl peptidase 4 OS=Oxyuranus scutellatus OX=8668 PE=2 SV=1 | Cellular components |
| 61 | 546 | tr\|A6MJH3\|A6MJH3_OXYMI | 125.79 | 4 | 4 | 3.22E+06 | 3 | 3 | 3 | Carbamidomethylation | 86590 | Dipeptidyl peptidase 4 OS=Oxyuranus microlepidotus OX=111177 PE=2 SV=1 | Cellular components |
| 57 | 444 | tr\|U3FAK1\|U3FAK1_MICFL | 121.14 | 9 | 9 | 9.41E+06 | 4 | 4 | 4 | Carbamidomethylation | 47564 | Vascular endothelial growth factor 2 OS=Micrurus fulvius OX=8637 PE=2 SV=1 | Venom endothelial growth factors |
| 57 | 470 | tr\|V8NCP7\|V8NCP7_OPHHA | 121.14 | 9 | 9 | 9.41E+06 | 4 | 4 | 4 | Carbamidomethylation | 47557 | Vascular endothelial growth factor C OS=Ophiophagus hannah OX=8665 GN=VEGFC PE=3 SV=1 | Venom endothelial growth factors |
| 57 | 471 | tr\|A0A098LX21\|A0A098LX21_PANGU | 121.14 | 9 | 9 | 9.41E+06 | 4 | 4 | 4 | Carbamidomethylation | 47507 | Vascular endothelial growth factor C OS=Pantherophis guttatus OX=94885 GN=VEGFC PE=2 SV=1 | Venom endothelial growth factors |
| 57 | 474 | tr\|A0A2D4LA60\|A0A2D4LA60_9SAUR | 121.14 | 8 | 8 | 9.41E+06 | 4 | 4 | 4 | Carbamidomethylation | 53456 | PDGF_2 domain-containing protein (Fragment) OS=Micrurus spixii OX=129469 PE=3 SV=1 | Venom endothelial growth factors |
| 47 | 834 | Q9DEQ3\|3SO8_NAJAT | 119.11 | 38 | 38 | 4.07E+07 | 5 | 2 | 8 | Carbamidomethylation | 9962 | Neurotoxin homolog NL1 OS=Naja atra OX=8656 PE=3 SV=1 | 3-finger toxins |
| 47 | 835 | Q9W727\|3SO8_BUNMU | 119.11 | 38 | 38 | 4.07E+07 | 5 | 2 | 8 | Carbamidomethylation | 9934 | Muscarinic toxin-like protein OS=Bungarus multicinctus OX=8616 PE=3 SV=1 | 3-finger toxins |
| 73 | 664 | tr\|A0A0B8RY19\|A0A0B8RY19_BOIIR | 110.67 | 9 | 9 | 1.75E+06 | 3 | 3 | 3 |  | 44109 | Glia-derived nexin-like protein OS=Boiga irregularis OX=92519 PE=3 SV=1 | Protein family not assigned |
| 60 | 873 | tr\|V8NEU2\|V8NEU2_OPHHA | 110.06 | 11 | 11 | 4.04E+07 | 3 | 2 | 3 |  | 24506 | B30.2/SPRY domain-containing protein OS=Ophiophagus hannah OX=8665 GN=L345_13461 PE=3 SV=1 | Cellular components |
| 56 | 246 | tr\|V8NSK8\|V8NSK8_OPHHA | 104.48 | 2 | 2 | 9.39E+05 | 2 | 2 | 2 | Carbamidomethylation | 105984 | Golgi apparatus protein 1 (Fragment) OS=Ophiophagus hannah OX=8665 GN=GLG1 PE=4 SV=1 | Cellular components |
| 56 | 245 | tr\|A0A2D4PZB5\|A0A2D4PZB5_MICSU | 104.48 | 2 | 2 | 9.39E+05 | 2 | 2 | 2 | Carbamidomethylation | 96178 | Golgi apparatus protein 1 OS=Micrurus surinamensis OX=129470 PE=4 SV=1 | Cellular components |
| 56 | 248 | tr\|A0A2D4PXP4\|A0A2D4PXP4_MICSU | 104.48 | 2 | 2 | 9.39E+05 | 2 | 2 | 2 | Carbamidomethylation | 122130 | Golgi apparatus protein 1 (Fragment) OS=Micrurus surinamensis OX=129470 PE=4 SV=1 | Cellular components |
| 94 | 1888 | tr\|U3FCT9\|U3FCT9_MICFL | 101.58 | 5 | 5 | 2.10E+07 | 2 | 2 | 2 |  | 31577 | Endonuclease domain-containing 1 protein OS=Micrurus fulvius OX=8637 PE=2 SV=1 | Cellular components |
| 49 | 715 | tr\|A0A346CIA6\|A0A346CIA6_9SAUR | 101.48 | 12 | 12 | 1.62E+07 | 4 | 2 | 5 | Carbamidomethylation | 26943 | Cysteine-rich secretory protein (Fragment) OS=Spilotes sulphureus OX=1899469 PE=2 SV=1 | Cysteine-rich venom proteins |
| 64 | 885 | Q10749\|VM3M1_NAJMO | 97.2 | 4 | 4 | 1.18E+06 | 3 | 1 | 3 |  | 68176 | Snake venom metalloproteinase-disintegrin-like mocarhagin OS=Naja mossambica OX=8644 PE=1 SV=3 | Snake venom metalloproteinases |
| 68 | 854 | tr\|V8NW35\|V8NW35_OPHHA | 96.46 | 4 | 4 | 3.35E+06 | 2 | 2 | 2 | Carbamidomethylation | 60759 | Macrophage colony-stimulating factor 1 OS=Ophiophagus hannah OX=8665 GN=Csf1 PE=4 SV=1 | Cellular components |
| 67 | 1199 | tr\|A0A2D4Q7C6\|A0A2D4Q7C6_MICSU | 92.98 | 15 | 15 | 8.22E+06 | 3 | 1 | 3 |  | 14772 | TED_complement domain-containing protein (Fragment) OS=Micrurus surinamensis OX=129470 PE=4 SV=1 | Cellular components |
| 83 | 1085 | tr\|A0A6J1VMA6\|A0A6J1VMA6_9SAUR | 92.1 | 4 | 4 | 1.06E+07 | 2 | 2 | 2 | Carbamidomethylation | 50196 | hepatocyte growth factor activator OS=Notechis scutatus OX=8663 GN=HGFAC PE=3 SV=1 | Cellular components |
| 83 | 1044 | tr\|V8NIX6\|V8NIX6_OPHHA | 92.1 | 6 | 6 | 1.06E+07 | 2 | 2 | 2 | Carbamidomethylation | 37257 | Coagulation factor XII (Fragment) OS=Ophiophagus hannah OX=8665 GN=F12 PE=4 SV=1 | Snake venom serine proteinases |
| 78 | 1412 | tr\|V8NBS6\|V8NBS6_OPHHA | 90.65 | 15 | 15 | 2.83E+07 | 1 | 1 | 1 |  | 9348 | Uncharacterized protein (Fragment) OS=Ophiophagus hannah OX=8665 GN=L345_15265 PE=4 SV=1 | Protein family not assigned |
| 65 | 720 | tr\|V8NRR7\|V8NRR7_OPHHA | 90.59 | 2 | 2 | 1.34E+07 | 2 | 2 | 2 |  | 100176 | 39S ribosomal protein L16 mitochondrial (Fragment) OS=Ophiophagus hannah OX=8665 GN=MRPL16 PE=3 SV=1 | Cellular components |
| 72 | 1159 | P01401\|3NO2B_NAJHH | 85.86 | 26 | 26 | 1.32E+08 | 2 | 2 | 2 | Carbamidomethylation | 7546 | Weak toxin CM-11 OS=Naja haje haje OX=8642 PE=1 SV=1 | 3-finger toxins |
| 89 | 432 | tr\|A0A0B8RQP5\|A0A0B8RQP5_BOIIR | 84.58 | 3 | 3 | 4.21E+06 | 2 | 2 | 2 | Carbamidomethylation | 64088 | Phospholipase B-like OS=Boiga irregularis OX=92519 PE=3 SV=1 | Phospholipases B |
| 89 | 159 | tr\|A0A898INS1\|A0A898INS1_CALBG | 84.58 | 3 | 3 | 4.21E+06 | 2 | 2 | 2 | Carbamidomethylation | 64048 | Phospholipase B OS=Calliophis bivirgatus OX=8633 PE=2 SV=1 | Phospholipases B |
| 90 | 1183 | tr\|A0A182C5R8\|A0A182C5R8_9SAUR | 84.42 | 13 | 13 | 1.22E+07 | 2 | 2 | 2 | Carbamidomethylation | 20534 | Papilin (Fragment) OS=Phalotris mertensi OX=1260334 PE=4 SV=1 | Venom Kunitz-type family |
| 77 | 860 | tr\|A0A0B8RSX4\|A0A0B8RSX4_BOIIR | 84.23 | 3 | 3 | 3.67E+06 | 2 | 1 | 2 |  | 68610 | Carboxylic ester hydrolase OS=Boiga irregularis OX=92519 PE=3 SV=1 | Cellular components |
| 77 | 867 | tr\|A0A346CLZ4\|A0A346CLZ4_9SAUR | 84.23 | 3 | 3 | 3.67E+06 | 2 | 1 | 2 |  | 68520 | Carboxylic ester hydrolase (Fragment) OS=Ahaetulla prasina OX=499056 PE=2 SV=1 | Cellular components |
| 66 | 485 | tr\|A0A346CM26\|A0A346CM26_9SAUR | 83.86 | 3 | 3 | 2.90E+06 | 2 | 1 | 2 | Carbamidomethylation | 71879 | Metalloproteinase 6 (Fragment) OS=Ahaetulla prasina OX=499056 PE=2 SV=1 | Snake venom metalloproteinases |
| 66 | 491 | tr\|A0A346CM07\|A0A346CM07_9SAUR | 83.86 | 3 | 3 | 2.90E+06 | 2 | 1 | 2 | Carbamidomethylation | 67772 | Metalloproteinase 15 (Fragment) OS=Ahaetulla prasina OX=499056 PE=2 SV=1 | Snake venom metalloproteinases |
| 66 | 490 | tr\|A0A346CM39\|A0A346CM39_9SAUR | 83.86 | 3 | 3 | 2.90E+06 | 2 | 1 | 2 | Carbamidomethylation | 67739 | Metalloproteinase 38 (Fragment) OS=Ahaetulla prasina OX=499056 PE=2 SV=1 | Snake venom metalloproteinases |
| 66 | 510 | tr\|A0A346CM33\|A0A346CM33_9SAUR | 83.86 | 3 | 3 | 2.90E+06 | 2 | 1 | 2 | Carbamidomethylation | 67851 | Metalloproteinase 27 (Fragment) OS=Ahaetulla prasina OX=499056 PE=2 SV=1 | Snake venom metalloproteinases |
| 71 | 1099 | tr\|A0A194AR88\|A0A194AR88_9SAUR | 81.81 | 14 | 14 | 1.13E+08 | 2 | 1 | 3 |  | 20929 | Vespryn OS=Micrurus tener OX=1114301 PE=3 SV=1 | Vespryns |
| 86 | 1192 | tr\|A0A2D4PI83\|A0A2D4PI83_MICSU | 81.79 | 8 | 8 | 3.12E+06 | 2 | 2 | 2 | Carbamidomethylation | 30373 | Uncharacterized protein (Fragment) OS=Micrurus surinamensis OX=129470 PE=3 SV=1 | Protein family not assigned |
| 86 | 1413 | tr\|A0A2D4ETG2\|A0A2D4ETG2_MICCO | 81.79 | 10 | 10 | 3.12E+06 | 2 | 2 | 2 | Carbamidomethylation | 26065 | Uncharacterized protein OS=Micrurus corallinus OX=54390 PE=3 SV=1 | Protein family not assigned |
| 86 | 1414 | tr\|A0A2D4PI88\|A0A2D4PI88_MICSU | 81.79 | 5 | 5 | 3.12E+06 | 2 | 2 | 2 | Carbamidomethylation | 47224 | Uncharacterized protein (Fragment) OS=Micrurus surinamensis OX=129470 PE=3 SV=1 | Protein family not assigned |
| 86 | 1415 | tr\|A0A2D4ETB9\|A0A2D4ETB9_MICCO | 81.79 | 4 | 4 | 3.12E+06 | 2 | 2 | 2 | Carbamidomethylation | 58521 | Uncharacterized protein OS=Micrurus corallinus OX=54390 PE=3 SV=1 | Protein family not assigned |
| 74 | 503 | tr\|U3FZS8\|U3FZS8_MICFL | 81.71 | 3 | 3 | 2.99E+06 | 3 | 3 | 3 | Carbamidomethylation | 112194 | Aminopeptidase OS=Micrurus fulvius OX=8637 PE=2 SV=1 | Aminopeptidases |
| 95 | 1622 | tr\|A0A6J1W9L1\|A0A6J1W9L1_9SAUR | 76.1 | 6 | 6 | 2.73E+07 | 1 | 1 | 1 |  | 21932 | cysteine-rich venom protein ophanin-like OS=Notechis scutatus OX=8663 GN=LOC113430936 PE=3 SV=1 | Cysteine-rich venom proteins |
| 115 | 1885 | tr\|A0A2D4GU19\|A0A2D4GU19_MICCO | 74.72 | 14 | 14 | 1.20E+07 | 1 | 1 | 1 | Carbamidomethylation | 11337 | Uncharacterized protein (Fragment) OS=Micrurus corallinus OX=54390 PE=3 SV=1 | Protein family not assigned |
| 79 | 1452 | tr\|V8P0T5\|V8P0T5_OPHHA | 72.73 | 7 | 7 | 3.89E+06 | 2 | 2 | 2 |  | 39348 | Tumor necrosis factor receptor superfamily member 11B OS=Ophiophagus hannah OX=8665 GN=TNFRSF11B PE=4 SV=1 | Cellular components |
| 88 | 1013 | tr\|A0A1W7RDU2\|A0A1W7RDU2_AGKCO | 72.67 | 16 | 16 | 1.45E+06 | 2 | 2 | 2 |  | 14756 | 60S ribosomal protein L40 OS=Agkistrodon contortrix contortrix OX=8713 PE=3 SV=1 | Cellular components |
| 88 | 1011 | tr\|A0A2D4GWT6\|A0A2D4GWT6_MICCO | 72.67 | 16 | 16 | 1.45E+06 | 2 | 2 | 2 |  | 14728 | 60S ribosomal protein L40 OS=Micrurus corallinus OX=54390 PE=3 SV=1 | Cellular components |
| 88 | 1012 | tr\|U3FZ24\|U3FZ24_MICFL | 72.67 | 16 | 16 | 1.45E+06 | 2 | 2 | 2 |  | 14716 | 60S ribosomal protein L40 OS=Micrurus fulvius OX=8637 PE=2 SV=1 | Cellular components |
| 88 | 1014 | tr\|U3FBE5\|U3FBE5_MICFL | 72.67 | 16 | 16 | 1.45E+06 | 2 | 2 | 2 |  | 14728 | 60S ribosomal protein L40 OS=Micrurus fulvius OX=8637 PE=2 SV=1 | Cellular components |
| 88 | 1015 | tr\|A0A0B8RXA8\|A0A0B8RXA8_BOIIR | 72.67 | 16 | 16 | 1.45E+06 | 2 | 2 | 2 |  | 14728 | 60S ribosomal protein L40 OS=Boiga irregularis OX=92519 PE=3 SV=1 | Cellular components |
| 88 | 1016 | tr\|J3S9G5\|J3S9G5_CROAD | 72.67 | 16 | 16 | 1.45E+06 | 2 | 2 | 2 |  | 14728 | 60S ribosomal protein L40 OS=Crotalus adamanteus OX=8729 PE=2 SV=1 | Cellular components |
| 88 | 1184 | tr\|A0A2D4NTF5\|A0A2D4NTF5_MICSU | 72.67 | 16 | 16 | 1.45E+06 | 2 | 2 | 2 |  | 14987 | Uncharacterized protein (Fragment) OS=Micrurus surinamensis OX=129470 PE=4 SV=1 | Protein family not assigned |
| 88 | 1140 | tr\|A0A1W7RDR4\|A0A1W7RDR4_AGKCO | 72.67 | 13 | 13 | 1.45E+06 | 2 | 2 | 2 |  | 17979 | 40S ribosomal protein S27a OS=Agkistrodon contortrix contortrix OX=8713 PE=3 SV=1 | Cellular components |
| 88 | 1141 | tr\|A0A2D4G0P2\|A0A2D4G0P2_MICCO | 72.67 | 13 | 13 | 1.45E+06 | 2 | 2 | 2 |  | 17979 | 40S ribosomal protein S27a OS=Micrurus corallinus OX=54390 PE=3 SV=1 | Cellular components |
| 88 | 1137 | tr\|A0A2D4N2W9\|A0A2D4N2W9_9SAUR | 72.67 | 13 | 13 | 1.45E+06 | 2 | 2 | 2 |  | 17979 | 40S ribosomal protein S27a OS=Micrurus spixii OX=129469 PE=3 SV=1 | Cellular components |
| 88 | 1138 | tr\|J3S0S4\|J3S0S4_CROAD | 72.67 | 13 | 13 | 1.45E+06 | 2 | 2 | 2 |  | 17979 | 40S ribosomal protein S27a OS=Crotalus adamanteus OX=8729 PE=2 SV=1 | Cellular components |
| 88 | 1142 | tr\|A0A0B8RWD5\|A0A0B8RWD5_BOIIR | 72.67 | 13 | 13 | 1.45E+06 | 2 | 2 | 2 |  | 17979 | 40S ribosomal protein S27a OS=Boiga irregularis OX=92519 PE=3 SV=1 | Cellular components |
| 88 | 1139 | tr\|U3F6K5\|U3F6K5_MICFL | 72.67 | 13 | 13 | 1.45E+06 | 2 | 2 | 2 |  | 17979 | 40S ribosomal protein S27a OS=Micrurus fulvius OX=8637 PE=2 SV=1 | Cellular components |
| 88 | 1143 | tr\|A0A2D4N0Y5\|A0A2D4N0Y5_9SAUR | 72.67 | 12 | 12 | 1.45E+06 | 2 | 2 | 2 |  | 19424 | 40S ribosomal protein S27a OS=Micrurus spixii OX=129469 PE=3 SV=1 | Cellular components |
| 88 | 1144 | tr\|A0A2D4G0L1\|A0A2D4G0L1_MICCO | 72.67 | 12 | 12 | 1.45E+06 | 2 | 2 | 2 |  | 20590 | 40S ribosomal protein S27a (Fragment) OS=Micrurus corallinus OX=54390 PE=3 SV=1 | Cellular components |
| 88 | 1185 | tr\|U3EQ35\|U3EQ35_MICFL | 72.67 | 6 | 6 | 1.45E+06 | 2 | 2 | 2 |  | 42865 | Polyubiquitin-C isoform 2 OS=Micrurus fulvius OX=8637 PE=2 SV=1 | Cellular components |
| 88 | 1107 | tr\|A0A2D4GX97\|A0A2D4GX97_MICCO | 72.67 | 17 | 17 | 1.45E+06 | 2 | 2 | 2 |  | 14220 | 60S ribosomal protein L40 OS=Micrurus corallinus OX=54390 PE=3 SV=1 | Cellular components |
| 88 | 1403 | tr\|A0A2D4PEV8\|A0A2D4PEV8_MICSU | 72.67 | 12 | 12 | 1.45E+06 | 2 | 2 | 2 |  | 20336 | Uncharacterized protein (Fragment) OS=Micrurus surinamensis OX=129470 PE=4 SV=1 | Cellular components |
| 75 | 791 | tr\|C6S3P7\|C6S3P7_DEIAC | 69.43 | 4 | 4 | 2.20E+07 | 2 | 2 | 2 |  | 51342 | Bactericidal/permeability-increasing protein-like 3 OS=Deinagkistrodon acutus OX=36307 GN=bpil3 PE=2 SV=1 | Cellular components |
| 119 | 1931 | tr\|V8PE21\|V8PE21_OPHHA | 68.01 | 6 | 6 | 4.09E+05 | 1 | 1 | 1 | Carbamidomethylation | 19894 | Endothelial cell-specific molecule 1 (Fragment) OS=Ophiophagus hannah OX=8665 GN=ESM1 PE=4 SV=1 | Cellular components |
| 85 | 916 | tr\|V8NCS7\|V8NCS7_OPHHA | 67.85 | 9 | 9 | 2.09E+07 | 2 | 2 | 2 |  | 24193 | Multiple inositol polyphosphate phosphatase 1 (Fragment) OS=Ophiophagus hannah OX=8665 GN=MINPP1 PE=4 SV=1 | Cellular components |
| 85 | 978 | tr\|A0A2D4MRZ8\|A0A2D4MRZ8_9SAUR | 67.85 | 6 | 6 | 2.09E+07 | 2 | 2 | 2 |  | 34621 | Uncharacterized protein (Fragment) OS=Micrurus spixii OX=129469 PE=4 SV=1 | Protein family not assigned |
| 110 | 1714 | tr\|A0A2D4MC13\|A0A2D4MC13_9SAUR | 66.57 | 3 | 3 | 5.77E+05 | 1 | 1 | 1 | Carbamidomethylation | 41305 | GH18 domain-containing protein OS=Micrurus spixii OX=129469 PE=4 SV=1 | Cellular components |
| 87 | 1557 | tr\|V8N885\|V8N885_OPHHA | 64.31 | 6 | 6 | 1.67E+06 | 1 | 1 | 1 |  | 18615 | NTR domain-containing protein (Fragment) OS=Ophiophagus hannah OX=8665 GN=L345_16505 PE=4 SV=1 | Cellular components |
| 87 | 2017 | tr\|A0A2D4ETJ5\|A0A2D4ETJ5_MICCO | 64.31 | 8 | 8 | 1.67E+06 | 1 | 1 | 1 |  | 13125 | NTR domain-containing protein (Fragment) OS=Micrurus corallinus OX=54390 PE=4 SV=1 | Cellular components |
| 107 | 1732 | Q2VBN8\|3SDC9_OPHHA | 62.31 | 10 | 10 | 3.41E+06 | 1 | 1 | 1 | Carbamidomethylation | 9380 | Beta-cardiotoxin CTX9 OS=Ophiophagus hannah OX=8665 PE=1 SV=1 | 3-finger toxins |
| 107 | 1733 | Q53B46\|3SDC5_OPHHA | 62.31 | 10 | 10 | 3.41E+06 | 1 | 1 | 1 | Carbamidomethylation | 9352 | Beta-cardiotoxin CTX15 OS=Ophiophagus hannah OX=8665 PE=1 SV=1 | 3-finger toxins |
| 107 | 1969 | tr\|V8N6H4\|V8N6H4_OPHHA | 62.31 | 11 | 11 | 3.41E+06 | 1 | 1 | 1 | Carbamidomethylation | 7965 | Uncharacterized protein (Fragment) OS=Ophiophagus hannah OX=8665 GN=L345_17130 PE=4 SV=1 | Protein family not assigned |
| 107 | 1974 | Q69CK0\|3SDC7_OPHHA | 62.31 | 10 | 10 | 3.41E+06 | 1 | 1 | 1 | Carbamidomethylation | 9311 | Beta-cardiotoxin CTX27 OS=Ophiophagus hannah OX=8665 PE=1 SV=1 | 3-finger toxins |
| 120 | 1929 | tr\|A0A898INR6\|A0A898INR6_CALBG | 61.92 | 9 | 9 | 0 | 1 | 1 | 1 | Carbamidomethylation | 16742 | Phospholipase A2 IB OS=Calliophis bivirgatus OX=8633 PE=2 SV=1 | Phospholipases A2 |
| 120 | 1839 | tr\|A0A898INH4\|A0A898INH4_CALBG | 61.92 | 9 | 9 | 0 | 1 | 1 | 1 | Carbamidomethylation | 16467 | Phospholipase A2 IB OS=Calliophis bivirgatus OX=8633 PE=2 SV=1 | Phospholipases A2 |
| 120 | 1840 | tr\|A0A898ILC3\|A0A898ILC3_CALBG | 61.92 | 9 | 9 | 0 | 1 | 1 | 1 | Carbamidomethylation | 16380 | Phospholipase A2 IB OS=Calliophis bivirgatus OX=8633 PE=2 SV=1 | Phospholipases A2 |
| 91 | 1127 | tr\|V8NH29\|V8NH29_OPHHA | 61.04 | 2 | 2 | 1.61E+06 | 1 | 1 | 1 |  | 54055 | Proactivator polypeptide OS=Ophiophagus hannah OX=8665 GN=PSAP PE=4 SV=1 | Cellular components |
| 121 | 1711 | tr\|V8P1S6\|V8P1S6_OPHHA | 60.91 | 7 | 7 | 4.67E+05 | 1 | 1 | 1 |  | 16451 | Ferritin OS=Ophiophagus hannah OX=8665 GN=FTH PE=3 SV=1 | Cellular components |
| 121 | 1939 | tr\|J3S4D4\|J3S4D4_CROAD | 60.91 | 5 | 5 | 4.67E+05 | 1 | 1 | 1 |  | 21252 | Ferritin OS=Crotalus adamanteus OX=8729 PE=2 SV=1 | Cellular components |
| 121 | 1640 | tr\|A0A2D4NEE8\|A0A2D4NEE8_9SAUR | 60.91 | 5 | 5 | 4.67E+05 | 1 | 1 | 1 |  | 21279 | Ferritin OS=Micrurus spixii OX=129469 PE=3 SV=1 | Cellular components |
| 121 | 1941 | tr\|U3FWZ4\|U3FWZ4_MICFL | 60.91 | 5 | 5 | 4.67E+05 | 1 | 1 | 1 |  | 21252 | Ferritin OS=Micrurus fulvius OX=8637 PE=2 SV=1 | Cellular components |
| 121 | 1940 | tr\|A0A0B8RYU5\|A0A0B8RYU5_BOIIR | 60.91 | 5 | 5 | 4.67E+05 | 1 | 1 | 1 |  | 21233 | Ferritin OS=Boiga irregularis OX=92519 PE=3 SV=1 | Cellular components |
| 121 | 1641 | tr\|A0A1W7RHC7\|A0A1W7RHC7_AGKCO | 60.91 | 5 | 5 | 4.67E+05 | 1 | 1 | 1 |  | 21252 | Ferritin OS=Agkistrodon contortrix contortrix OX=8713 PE=3 SV=1 | Cellular components |
| 100 | 59 | tr\|V8P2K1\|V8P2K1_OPHHA | 60.53 | 2 | 2 | 5.12E+05 | 1 | 1 | 1 |  | 91914 | Aminopeptidase (Fragment) OS=Ophiophagus hannah OX=8665 GN=ERAP1 PE=3 SV=1 | Aminopeptidases |
| 76 | 1554 | tr\|I6LJ76\|I6LJ76_AUSSU | 59.4 | 5 | 5 | 3.51E+07 | 1 | 1 | 1 |  | 18664 | C-type lectin galactose binding isoform OS=Austrelaps superbus OX=29156 PE=2 SV=1 | Snake venom metalloproteinases |
| 76 | 1746 | tr\|R4G314\|R4G314_9SAUR | 59.4 | 5 | 5 | 3.51E+07 | 1 | 1 | 1 |  | 18462 | LP-Pse-6 OS=Pseudonaja modesta OX=340912 PE=2 SV=1 | Snake venom metalloproteinases |
| 76 | 2033 | D2YVK1\|LECG_HOPST | 59.4 | 5 | 5 | 3.51E+07 | 1 | 1 | 1 |  | 18567 | C-type lectin galactose-binding isoform OS=Hoplocephalus stephensii OX=196418 PE=2 SV=1 | Snake venom metalloproteinases |
| 76 | 1673 | D2YVJ6\|LECG_PSEPO | 59.4 | 5 | 5 | 3.51E+07 | 1 | 1 | 1 |  | 18617 | C-type lectin galactose-binding isoform OS=Pseudechis porphyriacus OX=8671 PE=2 SV=1 | Snake venom metalloproteinases |
| 76 | 1674 | D2YVI2\|LECG_PSEAU | 59.4 | 5 | 5 | 3.51E+07 | 1 | 1 | 1 |  | 18687 | C-type lectin galactose-binding isoform OS=Pseudechis australis OX=8670 PE=1 SV=1 | Snake venom metalloproteinases |
| 76 | 2034 | tr\|D2YVL1\|D2YVL1_DEMVE | 59.4 | 5 | 5 | 3.51E+07 | 1 | 1 | 1 |  | 18508 | Venom C-type lectin galactose binding isoform variant 2 OS=Demansia vestigiata OX=412038 PE=2 SV=1 | Snake venom metalloproteinases |
| 76 | 1675 | Q90WI8\|LECG1_BUNFA | 59.4 | 5 | 5 | 3.51E+07 | 1 | 1 | 1 |  | 18638 | C-type lectin BfL-1 OS=Bungarus fasciatus OX=8613 PE=2 SV=1 | Snake venom metalloproteinases |
| 76 | 1807 | tr\|R4FIR9\|R4FIR9_9SAUR | 59.4 | 5 | 5 | 3.51E+07 | 1 | 1 | 1 |  | 18538 | LP-Pse-3 OS=Pseudonaja modesta OX=340912 PE=2 SV=1 | Snake venom metalloproteinases |
| 84 | 957 | tr\|A0A1W7RDK3\|A0A1W7RDK3_AGKCO | 59.15 | 2 | 2 | 3.91E+05 | 1 | 1 | 1 |  | 52770 | Methanethiol oxidase OS=Agkistrodon contortrix contortrix OX=8713 PE=3 SV=1 | Cellular components |
| 84 | 961 | tr\|A0A0B8RQ82\|A0A0B8RQ82_BOIIR | 59.15 | 2 | 2 | 3.91E+05 | 1 | 1 | 1 |  | 52805 | Methanethiol oxidase OS=Boiga irregularis OX=92519 PE=3 SV=1 | Cellular components |
| 84 | 960 | tr\|A0A2D4M6S7\|A0A2D4M6S7_9SAUR | 59.15 | 2 | 2 | 3.91E+05 | 1 | 1 | 1 |  | 52817 | Methanethiol oxidase OS=Micrurus spixii OX=129469 PE=3 SV=1 | Cellular components |
| 84 | 1475 | tr\|A0A2D4GUS2\|A0A2D4GUS2_MICCO | 59.15 | 3 | 3 | 3.91E+05 | 1 | 1 | 1 |  | 44312 | Methanethiol oxidase (Fragment) OS=Micrurus corallinus OX=54390 PE=3 SV=1 | Cellular components |
| 123 | 1976 | tr\|A0A182C5U2\|A0A182C5U2_9SAUR | 55.69 | 3 | 3 | 3.45E+06 | 1 | 1 | 1 | Carbamidomethylation | 33574 | Beta type PLA2 inhibitor (Fragment) OS=Phalotris mertensi OX=1260334 PE=4 SV=1 | Phospholipase inhibitors |
| 123 | 1977 | tr\|A0A346CI92\|A0A346CI92_9SAUR | 55.69 | 3 | 3 | 3.45E+06 | 1 | 1 | 1 | Carbamidomethylation | 36456 | Phospholipase inhibitor 1 (Fragment) OS=Spilotes sulphureus OX=1899469 PE=2 SV=1 | Phospholipase inhibitors |
| 123 | 1978 | tr\|A0A0B8RQP8\|A0A0B8RQP8_BOIIR | 55.69 | 3 | 3 | 3.45E+06 | 1 | 1 | 1 | Carbamidomethylation | 36722 | Phospholipase A2 inhibitor subunit B OS=Boiga irregularis OX=92519 PE=4 SV=1 | Phospholipase inhibitors |
| 122 | 1975 | tr\|A0A2D4N3K6\|A0A2D4N3K6_9SAUR | 53.47 | 10 | 10 | 3.83E+06 | 1 | 1 | 1 |  | 11252 | Pept_C1 domain-containing protein (Fragment) OS=Micrurus spixii OX=129469 PE=3 SV=1 | Snake venom serine proteinases |
| 105 | 1298 | tr\|A0A2D4FIT3\|A0A2D4FIT3_MICCO | 52.88 | 3 | 3 | 2.93E+06 | 1 | 1 | 1 | Carbamidomethylation | 39226 | Uncharacterized protein OS=Micrurus corallinus OX=54390 PE=4 SV=1 | Protein family not assigned |
| 105 | 1256 | tr\|V8NL73\|V8NL73_OPHHA | 52.88 | 3 | 3 | 2.93E+06 | 1 | 1 | 1 | Carbamidomethylation | 34271 | Extracellular matrix protein 1 (Fragment) OS=Ophiophagus hannah OX=8665 GN=Ecm1 PE=4 SV=1 | Cellular components |
| 102 | 1716 | tr\|V8PCI9\|V8PCI9_OPHHA | 51.51 | 1 | 1 | 9.60E+05 | 1 | 1 | 1 |  | 127525 | Peptidylamidoglycolate lyase (Fragment) OS=Ophiophagus hannah OX=8665 GN=Pam PE=4 SV=1 | Cellular components |
| 102 | 1988 | tr\|A0A2D4FSZ4\|A0A2D4FSZ4_MICCO | 51.51 | 2 | 2 | 9.60E+05 | 1 | 1 | 1 |  | 81636 | Uncharacterized protein (Fragment) OS=Micrurus corallinus OX=54390 PE=3 SV=1 | Protein family not assigned |
| 102 | 1989 | tr\|A0A2D4FTN3\|A0A2D4FTN3_MICCO | 51.51 | 2 | 2 | 9.60E+05 | 1 | 1 | 1 |  | 81707 | Uncharacterized protein (Fragment) OS=Micrurus corallinus OX=54390 PE=3 SV=1 | Protein family not assigned |
| 102 | 1990 | tr\|U3ERT9\|U3ERT9_MICFL | 51.51 | 1 | 1 | 9.60E+05 | 1 | 1 | 1 |  | 96651 | Peptidyl-glycine alpha-amidating monooxygenase OS=Micrurus fulvius OX=8637 PE=2 SV=1 | Cellular components |
| 102 | 1991 | tr\|A0A0B8RU52\|A0A0B8RU52_BOIIR | 51.51 | 1 | 1 | 9.60E+05 | 1 | 1 | 1 |  | 96865 | Peptidyl-glycine alpha-amidating monooxygenase OS=Boiga irregularis OX=92519 PE=3 SV=1 | Cellular components |
| 102 | 1987 | tr\|A0A2D4FT32\|A0A2D4FT32_MICCO | 51.51 | 2 | 2 | 9.60E+05 | 1 | 1 | 1 |  | 65984 | Uncharacterized protein (Fragment) OS=Micrurus corallinus OX=54390 PE=4 SV=1 | Protein family not assigned |
| 104 | 1735 | tr\|A0A6P9BFQ0\|A0A6P9BFQ0_PANGU | 51.15 | 4 | 4 | 1.31E+06 | 1 | 1 | 1 |  | 26901 | cationic trypsin-3-like isoform X1 OS=Pantherophis guttatus OX=94885 GN=LOC117661768 PE=3 SV=1 | Snake venom serine proteinases |
| 104 | 1736 | tr\|A0A6P9BES5\|A0A6P9BES5_PANGU | 51.15 | 4 | 4 | 1.31E+06 | 1 | 1 | 1 |  | 27050 | cationic trypsin-3-like isoform X2 OS=Pantherophis guttatus OX=94885 GN=LOC117661768 PE=3 SV=1 | Snake venom serine proteinases |
| 104 | 1549 | tr\|V8N4Z1\|V8N4Z1_OPHHA | 51.15 | 3 | 3 | 1.31E+06 | 1 | 1 | 1 |  | 34150 | Cationic trypsin-3 (Fragment) OS=Ophiophagus hannah OX=8665 GN=Try3 PE=3 SV=1 | Snake venom serine proteinases |
| 104 | 1548 | tr\|A0A6I9YQL3\|A0A6I9YQL3_9SAUR | 51.15 | 4 | 4 | 1.31E+06 | 1 | 1 | 1 |  | 26870 | cationic trypsin-3-like OS=Thamnophis sirtalis OX=35019 GN=LOC106552242 PE=3 SV=1 | Snake venom serine proteinases |
| 104 | 1982 | tr\|A0A670YWE2\|A0A670YWE2_PSETE | 51.15 | 4 | 4 | 1.31E+06 | 1 | 1 | 1 |  | 25367 | Peptidase S1 domain-containing protein OS=Pseudonaja textilis OX=8673 PE=3 SV=1 | Snake venom serine proteinases |
| 104 | 1984 | tr\|A0A6J1VIS1\|A0A6J1VIS1_9SAUR | 51.15 | 4 | 4 | 1.31E+06 | 1 | 1 | 1 |  | 26900 | cationic trypsin-3-like OS=Notechis scutatus OX=8663 GN=LOC113425235 PE=3 SV=1 | Snake venom serine proteinases |
| 104 | 1573 | tr\|A0A6I9YNT9\|A0A6I9YNT9_9SAUR | 51.15 | 4 | 4 | 1.31E+06 | 1 | 1 | 1 |  | 26778 | cationic trypsin-3-like OS=Thamnophis sirtalis OX=35019 GN=LOC106552243 PE=3 SV=1 | Snake venom serine proteinases |
| 104 | 1980 | tr\|A0A670JU78\|A0A670JU78_PODMU | 51.15 | 6 | 6 | 1.31E+06 | 1 | 1 | 1 |  | 17669 | Peptidase S1 domain-containing protein OS=Podarcis muralis OX=64176 PE=3 SV=1 | Snake venom serine proteinases |
| 104 | 1981 | tr\|A0A670K1A5\|A0A670K1A5_PODMU | 51.15 | 5 | 5 | 1.31E+06 | 1 | 1 | 1 |  | 20516 | Peptidase S1 domain-containing protein OS=Podarcis muralis OX=64176 PE=3 SV=1 | Snake venom serine proteinases |
| 104 | 1983 | tr\|A0A670JW80\|A0A670JW80_PODMU | 51.15 | 4 | 4 | 1.31E+06 | 1 | 1 | 1 |  | 25530 | Peptidase S1 domain-containing protein OS=Podarcis muralis OX=64176 PE=3 SV=1 | Snake venom serine proteinases |
| 104 | 1985 | tr\|A0A670JXY5\|A0A670JXY5_PODMU | 51.15 | 4 | 4 | 1.31E+06 | 1 | 1 | 1 |  | 28209 | Peptidase S1 domain-containing protein OS=Podarcis muralis OX=64176 PE=3 SV=1 | Snake venom serine proteinases |
| 104 | 1993 | tr\|A0A670JZC0\|A0A670JZC0_PODMU | 51.15 | 3 | 3 | 1.31E+06 | 1 | 1 | 1 |  | 39190 | Peptidase S1 domain-containing protein OS=Podarcis muralis OX=64176 PE=3 SV=1 | Snake venom serine proteinases |
| 108 | 1497 | tr\|A0A2D4PH67\|A0A2D4PH67_MICSU | 50.74 | 7 | 7 | 1.16E+07 | 1 | 1 | 1 |  | 17627 | Pept_C1 domain-containing protein (Fragment) OS=Micrurus surinamensis OX=129470 PE=3 SV=1 | Snake venom serine proteinases |
| 98 | 1485 | tr\|V8NCE3\|V8NCE3_OPHHA | 50.64 | 2 | 2 | 9.89E+06 | 1 | 1 | 1 |  | 51067 | Endonuclease domain-containing 1 protein (Fragment) OS=Ophiophagus hannah OX=8665 GN=ENDOD1 PE=4 SV=1 | Cellular components |
| 112 | 1108 | tr\|A0A0B8RZW3\|A0A0B8RZW3_BOIIR | 49.41 | 3 | 3 | 8.24E+05 | 1 | 1 | 1 |  | 52134 | Hyaluronidase OS=Boiga irregularis OX=92519 PE=3 SV=1 | Hyaluronidases |
| 112 | 773 | tr\|A0A898INC5\|A0A898INC5_CALBG | 49.41 | 3 | 3 | 8.24E+05 | 1 | 1 | 1 |  | 52241 | Hyaluronidase OS=Calliophis bivirgatus OX=8633 PE=2 SV=1 | Hyaluronidases |
| 112 | 931 | tr\|A0A077L860\|A0A077L860_PROFL | 49.41 | 2 | 2 | 8.24E+05 | 1 | 1 | 1 |  | 60400 | Hyaluronidase (Fragment) OS=Protobothrops flavoviridis OX=88087 PE=2 SV=1 | Hyaluronidases |
| 112 | 932 | tr\|U3TDE8\|U3TDE8_PROFL | 49.41 | 2 | 2 | 8.24E+05 | 1 | 1 | 1 |  | 61065 | Hyaluronidase (Fragment) OS=Protobothrops flavoviridis OX=88087 PE=2 SV=1 | Hyaluronidases |
| 99 | 1073 | tr\|A0A0B8RRE5\|A0A0B8RRE5_BOIIR | 46.55 | 2 | 2 | 1.17E+06 | 1 | 1 | 1 |  | 37567 | Cathepsin B OS=Boiga irregularis OX=92519 PE=3 SV=1 | Cathepsins |
| 99 | 1276 | tr\|U3FD65\|U3FD65_MICFL | 46.55 | 2 | 2 | 1.17E+06 | 1 | 1 | 1 |  | 37560 | Cathepsin B OS=Micrurus fulvius OX=8637 PE=2 SV=1 | Cathepsins |
| 99 | 1274 | tr\|A0A1W7RIP5\|A0A1W7RIP5_AGKCO | 46.55 | 2 | 2 | 1.17E+06 | 1 | 1 | 1 |  | 37351 | Cathepsin B OS=Agkistrodon contortrix contortrix OX=8713 PE=3 SV=1 | Cathepsins |
| 111 | 1502 | tr\|A0A6J1U723\|A0A6J1U723_9SAUR | 46.19 | 1 | 1 | 7.51E+05 | 1 | 1 | 1 | Carbamidomethylation | 75579 | Hepatocyte growth factor OS=Notechis scutatus OX=8663 GN=HGF PE=3 SV=1 | Cellular components |
| 111 | 1503 | tr\|A0A6J1U4Y2\|A0A6J1U4Y2_9SAUR | 46.19 | 1 | 1 | 7.51E+05 | 1 | 1 | 1 | Carbamidomethylation | 76111 | Hepatocyte growth factor OS=Notechis scutatus OX=8663 GN=HGF PE=3 SV=1 | Cellular components |
| 111 | 1540 | tr\|A0A670YMA3\|A0A670YMA3_PSETE | 46.19 | 1 | 1 | 7.51E+05 | 1 | 1 | 1 | Carbamidomethylation | 82067 | Hepatocyte growth factor OS=Pseudonaja textilis OX=8673 GN=HGF PE=3 SV=1 | Cellular components |
| 116 | 2011 | tr\|A0A2D4GN98\|A0A2D4GN98_MICCO | 45.49 | 8 | 8 | 1.02E+07 | 1 | 1 | 1 |  | 15362 | Uncharacterized protein (Fragment) OS=Micrurus corallinus OX=54390 PE=4 SV=1 | Protein family not assigned |
| 124 | 2040 | tr\|V8P8Z0\|V8P8Z0_OPHHA | 39.63 | 2 | 2 | 5.24E+05 | 1 | 1 | 1 |  | 60500 | Gamma-glutamyltranspeptidase 1 (Fragment) OS=Ophiophagus hannah OX=8665 GN=GGT1 PE=3 SV=1 | Cellular components |
| 114 | 2116 | tr\|V8N3T3\|V8N3T3_OPHHA | 39.17 | 5 | 5 | 1.10E+06 | 1 | 1 | 1 |  | 22732 | Pentaxin (Fragment) OS=Ophiophagus hannah OX=8665 GN=PTX2 PE=3 SV=1 | Cellular components |
| 114 | 2117 | tr\|A0A0B8RX63\|A0A0B8RX63_BOIIR | 39.17 | 4 | 4 | 1.10E+06 | 1 | 1 | 1 |  | 25320 | Pentaxin OS=Boiga irregularis OX=92519 PE=3 SV=1 | Cellular components |
| 113 | 1771 | tr\|A0A2D4NXP7\|A0A2D4NXP7_MICSU | 39 | 6 | 6 | 1.94E+06 | 1 | 1 | 1 |  | 16177 | Pentaxin (Fragment) OS=Micrurus surinamensis OX=129470 PE=3 SV=1 | Cellular components |
| 113 | 1543 | tr\|A0A2D4H2D0\|A0A2D4H2D0_MICCO | 39 | 6 | 6 | 1.94E+06 | 1 | 1 | 1 |  | 14737 | Uncharacterized protein (Fragment) OS=Micrurus corallinus OX=54390 PE=4 SV=1 | Protein family not assigned |
| 101 | 874 | tr\|T2HS00\|T2HS00_PROFL | 38.95 | 2 | 2 | 6.81E+05 | 1 | 1 | 1 | Carbamidomethylation | 58564 | Uncharacterized protein OS=Protobothrops flavoviridis OX=88087 PE=2 SV=1 | Protein family not assigned |
| 101 | 1079 | tr\|A0A0B8RXQ3\|A0A0B8RXQ3_BOIIR | 38.95 | 2 | 2 | 6.81E+05 | 1 | 1 | 1 | Carbamidomethylation | 58440 | Proactivator polypeptide-like OS=Boiga irregularis OX=92519 PE=4 SV=1 | Snake venom serine proteinases |
| 125 | 2144 | tr\|A0A2D4EV48\|A0A2D4EV48_MICCO | 38.93 | 7 | 7 | 1.31E+06 | 1 | 1 | 1 |  | 11941 | Ig-like domain-containing protein (Fragment) OS=Micrurus corallinus OX=54390 PE=4 SV=1 | Cellular components |
| 96 | 906 | tr\|V8P7T9\|V8P7T9_OPHHA | 38.71 | 1 | 1 | 1.53E+06 | 1 | 1 | 1 |  | 73994 | Sulfhydryl oxidase OS=Ophiophagus hannah OX=8665 GN=QSOX1 PE=3 SV=1 | Cellular components |
| 96 | 1284 | tr\|A0A1W7RCZ5\|A0A1W7RCZ5_AGKCO | 38.71 | 1 | 1 | 1.53E+06 | 1 | 1 | 1 |  | 88010 | Sulfhydryl oxidase OS=Agkistrodon contortrix contortrix OX=8713 PE=3 SV=1 | Cellular components |
