# Supplementary material for: Inhibition of bacterial biofilms by the snake venom proteome
Source: Biotechnol Rep (Amst). 2023 Aug 1;39:e00810. doi: 10.1016/j.btre.2023.e00810 (PMC10407894; doi:10.1016/j.btre.2023.e00810)
Supplement: Supplementary file 2 [file mmc2.docx]

Table S2. Proteomic data and protein family identification of *Bitis arietans* venom

| **Protein Group** | **Protein ID** | **Accession** | **-10lgP** | **Coverage (%)** | **Coverage (%) Sample 1** | **Area Sample 1** | **#Peptides** | **#Unique** | **#Spec Sample 1** | **Post Translation Modification** | **Average Mass** | **Description** | **Protein Family** |
| --- | --- | --- | --- | --- | --- | --- | --- | --- | --- | --- | --- | --- | --- |
| 3 | 212 | C0K3N1\|TXVE_BITAR | 245.99 | 46 | 46 | 7.84E+09 | 16 | 16 | 42 | Carbamidomethylation; Oxidation (M) | 16623 | Snake venom vascular endothelial growth factor toxin barietin OS=Bitis arietans OX=8692 PE=1 SV=1 | Venom endothelial growth factors |
| 2 | 90 | tr\|A0A5A4WNG2\|A0A5A4WNG2_BITAR | 268.21 | 56 | 56 | 8.59E+09 | 21 | 15 | 50 | Carbamidomethylation | 17812 | Bitiscetin-3 subunit alpha OS=Bitis arietans OX=8692 PE=2 SV=1 | C-lectin types |
| 1 | 60 | tr\|A0A5A4WN20\|A0A5A4WN20_BITAR | 255.11 | 69 | 69 | 1.35E+10 | 18 | 12 | 52 | Carbamidomethylation; Oxidation (M) | 17445 | Bitiscetin-3 subunit beta OS=Bitis arietans OX=8692 PE=2 SV=1 | C-lectin types |
| 18 | 532 | Q6T6T2\|DID2_BITGA | 228.72 | 48 | 48 | 8.43E+08 | 13 | 12 | 16 | Carbamidomethylation; Oxidation (M) | 13786 | Disintegrin gabonin-2 OS=Bitis gabonica OX=8694 PE=1 SV=1 | Disintegrins |
| 4 | 68 | tr\|D5KRX9\|D5KRX9_ECHOC | 247.54 | 38 | 38 | 4.12E+09 | 21 | 10 | 41 | Carbamidomethylation; Oxidation (M) | 29035 | Snake venom serine protease (Fragment) OS=Echis ocellatus OX=99586 PE=2 SV=1 | Snake venom serine proteinases |
| 123 | 59 | tr\|V8P2K1\|V8P2K1_OPHHA | 180.38 | 11 | 11 | 2.56E+07 | 8 | 8 | 8 | Carbamidomethylation | 91914 | Aminopeptidase (Fragment) OS=Ophiophagus hannah OX=8665 GN=ERAP1 PE=3 SV=1 | Aminopeptidases |
| 77 | 40 | tr\|T1D6P7\|T1D6P7_CROHD | 185.5 | 12 | 12 | 8.66E+07 | 10 | 8 | 10 | Carbamidomethylation | 96387 | Phosphodiesterase OS=Crotalus horridus OX=35024 PE=2 SV=1 | Phosphodiesterases |
| 5 | 121 | tr\|A0A6B7FPJ0\|A0A6B7FPJ0_VIPAA | 219.17 | 34 | 34 | 2.58E+09 | 13 | 8 | 29 | Carbamidomethylation | 28795 | Serine proteinase SP-8 OS=Vipera ammodytes ammodytes OX=8705 PE=2 SV=1 | Snake venom serine proteinases |
| 77 | 39 | P0DQQ4\|PDE_CRODO | 185.5 | 12 | 12 | 8.66E+07 | 10 | 8 | 10 | Carbamidomethylation | 94154 | Venom phosphodiesterase CdcPDE OS=Crotalus durissus collilineatus OX=221569 PE=1 SV=1 | Phosphodiesterases |
| 150 | 174 | tr\|V8ND68\|V8ND68_OPHHA | 156.51 | 13 | 13 | 5.56E+07 | 6 | 6 | 6 |  | 58279 | Phospholipase B-like (Fragment) OS=Ophiophagus hannah OX=8665 GN=PLBD1 PE=3 SV=1 | Phospholipases B |
| 22 | 201 | tr\|A0A2H4N3D5\|A0A2H4N3D5_BOTMO | 194.77 | 26 | 26 | 5.70E+08 | 8 | 4 | 18 | Carbamidomethylation; Oxidation (M) | 26837 | Cysteine-rich Venom Protein Moojin (Fragment) OS=Bothrops moojeni OX=98334 PE=2 SV=1 | Cysteine-rich venom proteins |
| 133 | 235 | tr\|A0A1W7REM3\|A0A1W7REM3_AGKCO | 162.35 | 22 | 22 | 1.22E+07 | 4 | 4 | 5 |  | 22832 | Peptidyl-prolyl cis-trans isomerase OS=Agkistrodon contortrix contortrix OX=8713 PE=3 SV=1 | Cellular components |
| 133 | 233 | tr\|A0A0B8RWS3\|A0A0B8RWS3_BOIIR | 162.35 | 22 | 22 | 1.22E+07 | 4 | 4 | 5 |  | 22790 | Peptidyl-prolyl cis-trans isomerase OS=Boiga irregularis OX=92519 PE=3 SV=1 | Cellular components |
| 133 | 236 | tr\|J3S474\|J3S474_CROAD | 162.35 | 22 | 22 | 1.22E+07 | 4 | 4 | 5 |  | 22853 | Peptidyl-prolyl cis-trans isomerase OS=Crotalus adamanteus OX=8729 PE=2 SV=1 | Cellular components |
| 133 | 234 | tr\|U3FWC8\|U3FWC8_MICFL | 162.35 | 22 | 22 | 1.22E+07 | 4 | 4 | 5 |  | 22829 | Peptidyl-prolyl cis-trans isomerase OS=Micrurus fulvius OX=8637 PE=2 SV=1 | Cellular components |
| 133 | 237 | tr\|U3F781\|U3F781_MICFL | 162.35 | 21 | 21 | 1.22E+07 | 4 | 4 | 5 |  | 24656 | Peptidyl-prolyl cis-trans isomerase OS=Micrurus fulvius OX=8637 PE=2 SV=1 | Cellular components |
| 149 | 1124 | tr\|Q6T7B8\|Q6T7B8_BITGA | 124.67 | 15 | 15 | 1.49E+08 | 4 | 4 | 5 | Carbamidomethylation | 15747 | Phospholipase A2-1 OS=Bitis gabonica OX=8694 PE=2 SV=1 | Phospholipases A2 |
| 62 | 644 | tr\|A0A077LA61\|A0A077LA61_PROEL | 182.29 | 17 | 17 | 9.64E+07 | 6 | 3 | 10 | Carbamidomethylation; Oxidation (M) | 26897 | Cysteine-rich venom protein OS=Protobothrops elegans OX=88086 PE=2 SV=1 | Cysteine-rich venom proteins |
| 183 | 858 | tr\|V8PGW5\|V8PGW5_OPHHA | 112.55 | 8 | 8 | 7.39E+06 | 3 | 3 | 3 | Carbamidomethylation | 45572 | Deoxyribonuclease II (Fragment) OS=Ophiophagus hannah OX=8665 GN=DNASE2 PE=3 SV=1 | Cellular components |
| 166 | 341 | A3QVN9\|HYAL1_BITAR | 130.06 | 8 | 8 | 2.06E+06 | 4 | 3 | 4 | Carbamidomethylation | 52312 | Hyaluronidase-1 OS=Bitis arietans OX=8692 PE=2 SV=1 | Hyaluronidases |
| 166 | 340 | A3QVP0\|HYAL2_BITAR | 130.06 | 8 | 8 | 2.06E+06 | 4 | 3 | 4 | Carbamidomethylation | 52255 | Hyaluronidase-2 OS=Bitis arietans OX=8692 PE=2 SV=1 | Hyaluronidases |
| 165 | 1362 | Q6T269\|VKT3_BITGA | 126.61 | 17 | 17 | 1.19E+08 | 4 | 3 | 5 | Carbamidomethylation | 16976 | Kunitz-type serine protease inhibitor bitisilin-3 (Fragment) OS=Bitis gabonica OX=8694 PE=2 SV=1 | Venom Kunitz-type family |
| 160 | 391 | tr\|A0A081DUA8\|A0A081DUA8_ECHCO | 123.21 | 14 | 14 | 1.03E+08 | 3 | 3 | 4 | Carbamidomethylation | 27186 | Nerve growth factor a OS=Echis coloratus OX=64175 PE=2 SV=1 | Nerve growth factors |
| 180 | 1067 | tr\|R4G7K2\|R4G7K2_9SAUR | 105.7 | 13 | 13 | 1.48E+08 | 2 | 2 | 2 | Carbamidomethylation | 26411 | CRiSP-Pse-11 OS=Pseudonaja modesta OX=340912 PE=2 SV=1 | Cysteine-rich venom proteins |
| 180 | 1380 | tr\|R4FIS5\|R4FIS5_9SAUR | 105.7 | 13 | 13 | 1.48E+08 | 2 | 2 | 2 | Carbamidomethylation | 26498 | CRiSP-Pse-17 OS=Pseudonaja modesta OX=340912 PE=2 SV=1 | Cysteine-rich venom proteins |
| 180 | 1381 | tr\|R4G2J3\|R4G2J3_9SAUR | 105.7 | 13 | 13 | 1.48E+08 | 2 | 2 | 2 | Carbamidomethylation | 26525 | CRiSP-Pse-4 OS=Pseudonaja modesta OX=340912 PE=2 SV=1 | Cysteine-rich venom proteins |
| 28 | 192 | tr\|A0A140DC06\|A0A140DC06_BITAR | 196.02 | 47 | 47 | 1.28E+08 | 8 | 2 | 14 | Carbamidomethylation | 17891 | C-type lectin 2 OS=Bitis arietans OX=8692 PE=2 SV=1 | C-lectin types |
| 180 | 1155 | tr\|A0A670YBA5\|A0A670YBA5_PSETE | 105.7 | 13 | 13 | 1.48E+08 | 2 | 2 | 2 | Carbamidomethylation | 25342 | Cysteine-rich venom protein pseudechetoxin-like OS=Pseudonaja textilis OX=8673 GN=LOC113444210 PE=3 SV=1 | Cysteine-rich venom proteins |
| 180 | 1156 | Q3SB05\|CRVP_PSETE | 105.7 | 13 | 13 | 1.48E+08 | 2 | 2 | 2 | Carbamidomethylation | 26429 | Cysteine-rich venom protein pseudechetoxin-like OS=Pseudonaja textilis OX=8673 PE=1 SV=1 | Cysteine-rich venom proteins |
| 88 | 595 | Q3BER3\|DID4A_ECHOC | 133.48 | 17 | 17 | 4.00E+09 | 3 | 2 | 7 | Carbamidomethylation | 13925 | Disintegrin EO4A OS=Echis ocellatus OX=99586 PE=1 SV=2 | Disintegrins |
| 88 | 529 | Q3BER4\|DID5A_ECHOC | 133.48 | 19 | 19 | 4.00E+09 | 3 | 2 | 7 | Carbamidomethylation | 12447 | Disintegrin EO5A OS=Echis ocellatus OX=99586 PE=1 SV=1 | Disintegrins |
| 20 | 12 | tr\|A0A0F7Z1K6\|A0A0F7Z1K6_CROAD | 239.95 | 22 | 22 | 2.83E+08 | 18 | 2 | 23 | Carbamidomethylation | 64682 | Ecto-5'-nucleotidase OS=Crotalus adamanteus OX=8729 PE=2 SV=1 | 5’-nucleotidase family |
| 20 | 14 | tr\|T1E3Y5\|T1E3Y5_CROHD | 239.95 | 22 | 22 | 2.83E+08 | 18 | 2 | 23 | Carbamidomethylation | 64817 | Ecto-5'-nucleotidase OS=Crotalus horridus OX=35024 PE=2 SV=1 | 5’-nucleotidase family |
| 20 | 17 | tr\|A0A194ASY3\|A0A194ASY3_SISMB | 239.95 | 22 | 22 | 2.83E+08 | 18 | 2 | 23 | Carbamidomethylation | 64944 | Ecto-5'-nucleotidase OS=Sistrurus miliarius barbouri OX=8759 PE=3 SV=1 | 5’-nucleotidase family |
| 20 | 10 | tr\|A0A194APR8\|A0A194APR8_SISTE | 239.95 | 22 | 22 | 2.83E+08 | 18 | 2 | 23 | Carbamidomethylation | 64651 | Ecto-5'-nucleotidase OS=Sistrurus tergeminus OX=8757 PE=3 SV=1 | 5’-nucleotidase family |
| 131 | 219 | tr\|E9KJY5\|E9KJY5_ECHOC | 145.11 | 6 | 6 | 1.32E+07 | 5 | 2 | 6 | Carbamidomethylation | 69422 | Group III snake venom metalloproteinase OS=Echis ocellatus OX=99586 GN=Eoc00024 PE=2 SV=1 | Snake venom metalloproteinases |
| 131 | 221 | tr\|E9KJY9\|E9KJY9_ECHOC | 145.11 | 6 | 6 | 1.32E+07 | 5 | 2 | 6 | Carbamidomethylation | 69449 | Group III snake venom metalloproteinase OS=Echis ocellatus OX=99586 GN=Eoc00024 PE=2 SV=1 | Snake venom metalloproteinases |
| 131 | 272 | tr\|E9KJY8\|E9KJY8_ECHOC | 145.11 | 6 | 6 | 1.32E+07 | 5 | 2 | 6 | Carbamidomethylation | 69515 | Group III snake venom metalloproteinase OS=Echis ocellatus OX=99586 GN=Eoc00024 PE=2 SV=1 | Snake venom metalloproteinases |
| 131 | 220 | tr\|Q2UXQ4\|Q2UXQ4_ECHOC | 145.11 | 6 | 6 | 1.32E+07 | 5 | 2 | 6 | Carbamidomethylation | 69470 | Group III snake venom metalloproteinse OS=Echis ocellatus OX=99586 GN=Svmp3-Eoc24 PE=2 SV=1 | Snake venom metalloproteinases |
| 47 | 251 | tr\|E9JG58\|E9JG58_ECHCO | 175.33 | 10 | 10 | 6.12E+06 | 8 | 2 | 10 | Carbamidomethylation | 56407 | Metalloproteinase (Fragment) OS=Echis coloratus OX=64175 PE=2 SV=1 | Snake venom metalloproteinases |
| 154 | 1043 | tr\|A0A0B8RYU4\|A0A0B8RYU4_BOIIR | 105.64 | 2 | 2 | 5.90E+07 | 2 | 2 | 4 | Carbamidomethylation | 69389 | Metalloproteinase (Type III) 3a OS=Boiga irregularis OX=92519 PE=4 SV=1 | Snake venom metalloproteinases |
| 154 | 1069 | tr\|A0A0B8RV81\|A0A0B8RV81_BOIIR | 105.64 | 2 | 2 | 5.90E+07 | 2 | 2 | 4 | Carbamidomethylation | 69417 | Metalloproteinase (Type III) 3b OS=Boiga irregularis OX=92519 PE=4 SV=1 | Snake venom metalloproteinases |
| 154 | 1068 | tr\|A0A0B8RRY8\|A0A0B8RRY8_BOIIR | 105.64 | 2 | 2 | 5.90E+07 | 2 | 2 | 4 | Carbamidomethylation | 69374 | Metalloproteinase (Type III) 3d OS=Boiga irregularis OX=92519 PE=4 SV=1 | Snake venom metalloproteinases |
| 92 | 277 | tr\|A0A194ARI7\|A0A194ARI7_SISTE | 156.71 | 12 | 12 | 1.47E+08 | 7 | 2 | 8 | Carbamidomethylation | 69092 | Metalloproteinase type III 9a OS=Sistrurus tergeminus OX=8757 PE=4 SV=1 | Snake venom metalloproteinases |
| 92 | 271 | tr\|A0A194APM5\|A0A194APM5_SISTE | 156.71 | 12 | 12 | 1.47E+08 | 7 | 2 | 8 | Carbamidomethylation | 69075 | Metalloproteinase type III 9b OS=Sistrurus tergeminus OX=8757 PE=4 SV=1 | Snake venom metalloproteinases |
| 65 | 335 | E0Y418\|VSP1_MACLB | 157.71 | 18 | 18 | 3.76E+07 | 6 | 2 | 10 | Carbamidomethylation; Oxidation (M) | 28702 | Serine protease VLSP-1 OS=Macrovipera lebetina OX=8709 PE=2 SV=1 | Snake venom serine proteinases |
| 109 | 226 | Q7LZK5\|SLA_BITAR | 144.24 | 30 | 30 | 6.84E+06 | 7 | 2 | 8 | Carbamidomethylation | 14935 | Snaclec bitiscetin subunit alpha OS=Bitis arietans OX=8692 PE=1 SV=1 | C-lectin types |
| 33 | 337 | Q7LZK8\|SLB_BITAR | 181.81 | 34 | 34 | 8.24E+08 | 7 | 2 | 10 | Carbamidomethylation | 14798 | Snaclec bitiscetin subunit beta OS=Bitis arietans OX=8692 PE=1 SV=1 | C-lectin types |
| 20 | 13 | F8S0Z7\|V5NTD_CROAD | 239.95 | 22 | 22 | 2.83E+08 | 18 | 2 | 23 | Carbamidomethylation | 64682 | Snake venom 5'-nucleotidase OS=Crotalus adamanteus OX=8729 PE=1 SV=2 | 5’-nucleotidase family |
| 189 | 874 | tr\|T2HS00\|T2HS00_PROFL | 78.81 | 3 | 3 | 1.47E+06 | 2 | 2 | 2 |  | 58564 | Uncharacterized protein OS=Protobothrops flavoviridis OX=88087 PE=2 SV=1 | Protein family not assigned |
| 208 | 1125 | tr\|A0A1W7RH08\|A0A1W7RH08_AGKCO | 64.46 | 2 | 2 | 1.17E+06 | 1 | 1 | 1 |  | 72067 | 78 kDa glucose-regulated protein OS=Agkistrodon contortrix contortrix OX=8713 PE=3 SV=1 | Cellular components |
| 208 | 1217 | tr\|A0A0B8RUJ6\|A0A0B8RUJ6_BOIIR | 64.46 | 2 | 2 | 1.17E+06 | 1 | 1 | 1 |  | 71941 | 78 kDa glucose-regulated protein OS=Boiga irregularis OX=92519 PE=3 SV=1 | Cellular components |
| 208 | 1126 | tr\|J3S4I0\|J3S4I0_CROAD | 64.46 | 2 | 2 | 1.17E+06 | 1 | 1 | 1 |  | 72105 | 78 kDa glucose-regulated protein OS=Crotalus adamanteus OX=8729 PE=2 SV=1 | Cellular components |
| 208 | 1149 | tr\|U3FCL7\|U3FCL7_MICFL | 64.46 | 2 | 2 | 1.17E+06 | 1 | 1 | 1 |  | 72106 | 78 kDa glucose-regulated protein OS=Micrurus fulvius OX=8637 PE=2 SV=1 | Cellular components |
| 208 | 1165 | tr\|V8NEC1\|V8NEC1_OPHHA | 64.46 | 2 | 2 | 1.17E+06 | 1 | 1 | 1 |  | 67365 | 78 kDa glucose-regulated protein OS=Ophiophagus hannah OX=8665 GN=HSPA5 PE=3 SV=1 | Cellular components |
| 173 | 809 | P86389\|PA2A2_BOTAS | 75.62 | 6 | 6 | 6.08E+08 | 1 | 1 | 1 | Carbamidomethylation | 14194 | Acidic phospholipase A2 2 OS=Bothrops asper OX=8722 PE=1 SV=1 | Phospholipases A2 |
| 173 | 878 | Q7T3T5\|PA2AB_DABSI | 75.62 | 5 | 5 | 6.08E+08 | 1 | 1 | 1 | Carbamidomethylation | 14828 | Acidic phospholipase A2 daboiatoxin B chain (Fragment) OS=Daboia siamensis OX=343250 PE=1 SV=1 | Phospholipases A3 |
| 6 | 28 | tr\|A0A2H4N3C7\|A0A2H4N3C7_BOTMO | 240.75 | 18 | 18 | 4.34E+07 | 18 | 1 | 26 | Carbamidomethylation; Oxidation (M) | 110032 | Aminopeptidase (Fragment) OS=Bothrops moojeni OX=98334 PE=2 SV=1 | Aminopeptidases |
| 7 | 25 | tr\|A5HUI5\|A5HUI5_GLOBR | 239.73 | 19 | 19 | 4.88E+07 | 18 | 1 | 26 | Carbamidomethylation; Oxidation (M) | 110153 | Aminopeptidase OS=Gloydius brevicaudus OX=259325 PE=2 SV=1 | Aminopeptidases |
| 210 | 2036 | tr\|A0A2D4ETZ6\|A0A2D4ETZ6_MICCO | 46.55 | 4 | 4 | 6.84E+05 | 1 | 1 | 1 |  | 26275 | ANTH domain-containing protein (Fragment) OS=Micrurus corallinus OX=54390 PE=4 SV=1 | Cellular components |
| 210 | 1788 | tr\|A0A2D4ETY6\|A0A2D4ETY6_MICCO | 46.55 | 2 | 2 | 6.84E+05 | 1 | 1 | 1 |  | 48221 | ANTH domain-containing protein (Fragment) OS=Micrurus corallinus OX=54390 PE=4 SV=1 | Cellular components |
| 210 | 1790 | tr\|A0A2D4ETX7\|A0A2D4ETX7_MICCO | 46.55 | 2 | 2 | 6.84E+05 | 1 | 1 | 1 |  | 53420 | ANTH domain-containing protein (Fragment) OS=Micrurus corallinus OX=54390 PE=4 SV=1 | Cellular components |
| 191 | 1473 | Q10755\|PA2B_VIPAZ | 60.41 | 6 | 6 | 3.98E+05 | 1 | 1 | 1 | Carbamidomethylation | 13856 | Basic phospholipase A2 B chain OS=Vipera aspis zinnikeri OX=55427 PE=1 SV=1 | Phospholipases A2 |
| 191 | 1474 | P0DKR5\|PA2B2_PSEFE | 60.41 | 6 | 6 | 3.98E+05 | 1 | 1 | 1 | Carbamidomethylation | 13735 | Basic phospholipase A2 CbII OS=Pseudocerastes fieldi OX=1355908 PE=1 SV=1 | Phospholipases A2 |
| 191 | 29670 | Q1RP78\|PA2B2_VIPBN | 60.41 | 5 | 5 | 3.98E+05 | 1 | 1 | 1 | Carbamidomethylation | 15594 | Basic phospholipase A2 chain HDP-2P OS=Vipera berus nikolskii OX=1808362 PE=1 SV=1 | Phospholipases A2 |
| 191 | 29671 | Q8JFG0\|PA2B_VIPAP | 60.41 | 5 | 5 | 3.98E+05 | 1 | 1 | 1 | Carbamidomethylation | 15610 | Basic phospholipase A2 vaspin B chain OS=Vipera aspis aspis OX=194601 PE=2 SV=1 | Phospholipases A2 |
| 191 | 29669 | P14420\|PA2B_VIPAE | 60.41 | 6 | 6 | 3.98E+05 | 1 | 1 | 1 | Carbamidomethylation | 13828 | Basic phospholipase A2 vipoxin B chain OS=Vipera ammodytes meridionalis OX=73841 PE=1 SV=1 | Phospholipases A2 |
| 187 | 1080 | tr\|A0A1L8D610\|A0A1L8D610_BOTAT | 78.92 | 9 | 9 | 2.84E+07 | 2 | 1 | 2 | Carbamidomethylation | 28380 | BATXSVSP11 OS=Bothrops atrox OX=8725 PE=2 SV=1 | Snake venom serine proteinases |
| 143 | 515 | E0Y419\|VSPBF_MACLB | 110.71 | 12 | 12 | 1.17E+07 | 4 | 1 | 5 | Carbamidomethylation; Oxidation (M) | 28297 | Beta-fibrinogenase OS=Macrovipera lebetina OX=8709 PE=1 SV=1 | Snake venom serine proteinases |
| 206 | 1549 | tr\|V8N4Z1\|V8N4Z1_OPHHA | 50.47 | 3 | 3 | 3.14E+06 | 1 | 1 | 1 |  | 34150 | Cationic trypsin-3 (Fragment) OS=Ophiophagus hannah OX=8665 GN=Try3 PE=3 SV=1 | Snake venom serine proteinases |
| 206 | 1735 | tr\|A0A6P9BFQ0\|A0A6P9BFQ0_PANGU | 50.47 | 4 | 4 | 3.14E+06 | 1 | 1 | 1 |  | 26901 | cationic trypsin-3-like isoform X1 OS=Pantherophis guttatus OX=94885 GN=LOC117661768 PE=3 SV=1 | Snake venom serine proteinases |
| 206 | 1736 | tr\|A0A6P9BES5\|A0A6P9BES5_PANGU | 50.47 | 4 | 4 | 3.14E+06 | 1 | 1 | 1 |  | 27050 | cationic trypsin-3-like isoform X2 OS=Pantherophis guttatus OX=94885 GN=LOC117661768 PE=3 SV=1 | Snake venom serine proteinases |
| 206 | 1984 | tr\|A0A6J1VIS1\|A0A6J1VIS1_9SAUR | 50.47 | 4 | 4 | 3.14E+06 | 1 | 1 | 1 |  | 26900 | cationic trypsin-3-like OS=Notechis scutatus OX=8663 GN=LOC113425235 PE=3 SV=1 | Snake venom serine proteinases |
| 206 | 1548 | tr\|A0A6I9YQL3\|A0A6I9YQL3_9SAUR | 50.47 | 4 | 4 | 3.14E+06 | 1 | 1 | 1 |  | 26870 | cationic trypsin-3-like OS=Thamnophis sirtalis OX=35019 GN=LOC106552242 PE=3 SV=1 | Snake venom serine proteinases |
| 206 | 1573 | tr\|A0A6I9YNT9\|A0A6I9YNT9_9SAUR | 50.47 | 4 | 4 | 3.14E+06 | 1 | 1 | 1 |  | 26778 | cationic trypsin-3-like OS=Thamnophis sirtalis OX=35019 GN=LOC106552243 PE=3 SV=1 | Snake venom serine proteinases |
| 214 | 1798 | tr\|V8P9K5\|V8P9K5_OPHHA | 55.89 | 2 | 2 | 8.57E+06 | 1 | 1 | 1 |  | 43993 | Counting factor associated protein D (Fragment) OS=Ophiophagus hannah OX=8665 GN=cfaD PE=3 SV=1 | Cellular components |
| 207 | 1747 | Q6T7B7\|LEC1_BITGA | 45.28 | 7 | 7 | 1.93E+06 | 1 | 1 | 1 | Carbamidomethylation | 18626 | C-type lectin 1 OS=Bitis gabonica OX=8694 PE=2 SV=1 | C-lectin types |
| 192 | 1241 | tr\|A0A194ARX7\|A0A194ARX7_SISTE | 75.23 | 9 | 9 | 1.01E+06 | 2 | 1 | 2 |  | 17989 | C-type lectin 15a OS=Sistrurus tergeminus OX=8757 PE=4 SV=1 | C-lectin types |
| 192 | 1240 | tr\|A0A194ATM7\|A0A194ATM7_SISTE | 75.23 | 9 | 9 | 1.01E+06 | 2 | 1 | 2 |  | 18005 | C-type lectin 15b OS=Sistrurus tergeminus OX=8757 PE=4 SV=1 | C-lectin types |
| 192 | 1417 | tr\|T1DH34\|T1DH34_CROHD | 75.23 | 10 | 10 | 1.01E+06 | 2 | 1 | 2 |  | 17911 | C-type lectin 19a OS=Crotalus horridus OX=35024 PE=2 SV=1 | C-lectin types |
| 192 | 1416 | tr\|A0A0K8RYZ2\|A0A0K8RYZ2_CROHD | 75.23 | 10 | 10 | 1.01E+06 | 2 | 1 | 2 |  | 17941 | C-type lectin 19b OS=Crotalus horridus OX=35024 PE=4 SV=1 | C-lectin types |
| 66 | 339 | tr\|A0A140DC05\|A0A140DC05_BITAR | 149.87 | 28 | 28 | 1.18E+07 | 6 | 1 | 7 | Carbamidomethylation | 17096 | C-type lectin 1b OS=Bitis arietans OX=8692 PE=2 SV=1 | C-lectin types |
| 192 | 1418 | tr\|A0A0F7Z2Q6\|A0A0F7Z2Q6_CROAD | 75.23 | 10 | 10 | 1.01E+06 | 2 | 1 | 2 |  | 17904 | C-type lectin 5 OS=Crotalus adamanteus OX=8729 PE=2 SV=1 | C-lectin types |
| 192 | 1421 | tr\|A0A194ASZ2\|A0A194ASZ2_SISMB | 75.23 | 9 | 9 | 1.01E+06 | 2 | 1 | 2 |  | 18073 | C-type lectin 8a OS=Sistrurus miliarius barbouri OX=8759 PE=4 SV=1 | C-lectin types |
| 192 | 1420 | tr\|A0A194AS79\|A0A194AS79_SISMB | 75.23 | 9 | 9 | 1.01E+06 | 2 | 1 | 2 |  | 18047 | C-type lectin 8b OS=Sistrurus miliarius barbouri OX=8759 PE=4 SV=1 | C-lectin types |
| 192 | 1422 | tr\|A0A194ATU4\|A0A194ATU4_SISMB | 75.23 | 9 | 9 | 1.01E+06 | 2 | 1 | 2 |  | 19656 | C-type lectin 8c OS=Sistrurus miliarius barbouri OX=8759 PE=4 SV=1 | C-lectin types |
| 207 | 2045 | P83519\|LECG_BOTJR | 45.28 | 7 | 7 | 1.93E+06 | 1 | 1 | 1 | Carbamidomethylation | 18653 | C-type lectin BJcuL OS=Bothrops jararacussu OX=8726 PE=1 SV=2 | Cellular components |
| 207 | 1760 | P0DL30\|LECG_BOTPI | 45.28 | 8 | 8 | 1.93E+06 | 1 | 1 | 1 | Carbamidomethylation | 16117 | C-type lectin BPL OS=Bothrops pirajai OX=113192 PE=1 SV=1 | C-lectin types |
| 207 | 2042 | P86970\|LECG_BOTPA | 45.28 | 8 | 8 | 1.93E+06 | 1 | 1 | 1 | Carbamidomethylation | 16223 | C-type lectin BpLec OS=Bothrops pauloensis OX=1042543 PE=1 SV=2 | C-lectin types |
| 21 | 156 | tr\|A0A1B3AXS3\|A0A1B3AXS3_BITAR | 214.63 | 42 | 42 | 1.16E+09 | 7 | 1 | 13 | Carbamidomethylation | 17943 | C-type lectin-like protein OS=Bitis arietans OX=8692 PE=2 SV=1 | C-lectin types |
| 198 | 1499 | tr\|A0A182C5Z6\|A0A182C5Z6_9SAUR | 68.09 | 7 | 7 | 4.47E+07 | 1 | 1 | 1 |  | 15505 | Cystatin OS=Phalotris mertensi OX=1260334 PE=3 SV=1 | Cystatins |
| 79 | 483 | tr\|A0A194APP6\|A0A194APP6_9SAUR | 141.62 | 20 | 20 | 0 | 4 | 1 | 11 | Oxidation (M) | 26681 | Cysteine-rich secretory protein 1 OS=Agkistrodon piscivorus OX=8715 PE=3 SV=1 | Cysteine-rich venom proteins |
| 79 | 472 | tr\|A0A194AMN4\|A0A194AMN4_9SAUR | 141.62 | 20 | 20 | 0 | 4 | 1 | 11 | Oxidation (M) | 26681 | Cysteine-rich secretory protein 2 OS=Agkistrodon piscivorus OX=8715 PE=3 SV=1 | Cysteine-rich venom proteins |
| 79 | 473 | Q7ZTA0\|CRVP_AGKPI | 141.62 | 20 | 20 | 0 | 4 | 1 | 11 | Oxidation (M) | 26681 | Cysteine-rich venom protein piscivorin OS=Agkistrodon piscivorus piscivorus OX=8716 PE=1 SV=1 | Cysteine-rich venom proteins |
| 175 | 153 | P01441\|3SA2_NAJOX | 92.47 | 20 | 20 | 2.78E+06 | 1 | 1 | 1 |  | 6636 | Cytotoxin 2 OS=Naja oxiana OX=8657 PE=1 SV=1 | 3-Finger toxins |
| 175 | 109 | Q9PS34\|3SA5_NAJOX | 92.47 | 20 | 20 | 2.78E+06 | 1 | 1 | 1 |  | 6664 | Cytotoxin Vc-5 OS=Naja oxiana OX=8657 PE=1 SV=1 | 3-Finger toxins |
| 182 | 1204 | P17497\|VM2_BITAR | 97.81 | 30 | 30 | 1.25E+08 | 2 | 1 | 3 | Carbamidomethylation | 9004 | Disintegrin bitistatin OS=Bitis arietans OX=8692 PE=1 SV=1 | Disintegrins |
| 38 | 26 | tr\|A0A182C5T8\|A0A182C5T8_9SAUR | 205.73 | 19 | 19 | 6.74E+06 | 15 | 1 | 17 | Carbamidomethylation | 57584 | Ecto-5'-nucleotidase (Fragment) OS=Phalotris mertensi OX=1260334 PE=3 SV=1 | 5’-nucleotidase family |
| 210 | 1627 | tr\|A0A2D4ETX9\|A0A2D4ETX9_MICCO | 46.55 | 2 | 2 | 6.84E+05 | 1 | 1 | 1 |  | 61362 | ENTH domain-containing protein (Fragment) OS=Micrurus corallinus OX=54390 PE=3 SV=1 | Cellular components |
| 210 | 1628 | tr\|A0A2D4ETW2\|A0A2D4ETW2_MICCO | 46.55 | 2 | 2 | 6.84E+05 | 1 | 1 | 1 |  | 64925 | ENTH domain-containing protein (Fragment) OS=Micrurus corallinus OX=54390 PE=3 SV=1 | Cellular components |
| 207 | 2043 | tr\|T2HS62\|T2HS62_OVOOK | 45.28 | 7 | 7 | 1.93E+06 | 1 | 1 | 1 | Carbamidomethylation | 18492 | Galactose binding lectin OS=Ovophis okinavensis OX=8769 PE=2 SV=1 | Cellular components |
| 197 | 1410 | tr\|A0A1S5QJJ2\|A0A1S5QJJ2_VIPAA | 80.95 | 23 | 23 | 3.65E+06 | 2 | 1 | 2 | Carbamidomethylation | 10312 | Kunitz/BPTI inhibitor-1 OS=Vipera ammodytes ammodytes OX=8705 PE=3 SV=1 | Venom Kunitz-type family |
| 197 | 1411 | P00992\|VKT3_VIPAA | 80.95 | 23 | 23 | 3.65E+06 | 2 | 1 | 2 | Carbamidomethylation | 10330 | Kunitz-type serine protease inhibitor 3 OS=Vipera ammodytes ammodytes OX=8705 PE=1 SV=2 | Venom Kunitz-type family |
| 181 | 1518 | Q6T6T5\|VKT1_BITGA | 65.99 | 9 | 9 | 1.50E+08 | 1 | 1 | 2 | Carbamidomethylation; Oxidation (M) | 9923 | Kunitz-type serine protease inhibitor bitisilin-1 OS=Bitis gabonica OX=8694 PE=1 SV=1 | Venom Kunitz-type family |
| 184 | 1494 | Q6T6S5\|VKT2_BITGA | 84.1 | 9 | 9 | 6.66E+07 | 1 | 1 | 1 |  | 10007 | Kunitz-type serine protease inhibitor bitisilin-2 OS=Bitis gabonica OX=8694 PE=1 SV=1 | Venom Kunitz-type family |
| 98 | 276 | tr\|A0A077LA85\|A0A077LA85_PROFL | 165.98 | 13 | 13 | 1.09E+06 | 7 | 1 | 8 | Carbamidomethylation | 58137 | Metalloprotease P-IIa 1 (Fragment) OS=Protobothrops flavoviridis OX=88087 PE=2 SV=1 | Snake venom metalloproteinases |
| 103 | 157 | tr\|E9JGD9\|E9JGD9_ECHCO | 177.04 | 15 | 15 | 1.19E+07 | 8 | 1 | 8 | Carbamidomethylation | 53290 | Metalloproteinase (Fragment) OS=Echis coloratus OX=64175 PE=2 SV=1 | Snake venom metalloproteinases |
| 54 | 188 | tr\|A0A0K8RZ04\|A0A0K8RZ04_CROHD | 183.33 | 12 | 12 | 4.93E+07 | 10 | 1 | 11 | Carbamidomethylation | 68342 | Metalloproteinase (Type III) 2b OS=Crotalus horridus OX=35024 PE=4 SV=1 | Snake venom metalloproteinases |
| 158 | 290 | tr\|E9JG52\|E9JG52_ECHCS | 135.32 | 6 | 6 | 3.87E+05 | 5 | 1 | 5 | Carbamidomethylation | 69351 | Metalloproteinase OS=Echis carinatus sochureki OX=124223 PE=2 SV=1 | Snake venom metalloproteinases |
| 158 | 291 | tr\|E9JG33\|E9JG33_ECHCS | 135.32 | 6 | 6 | 3.87E+05 | 5 | 1 | 5 | Carbamidomethylation | 69371 | Metalloproteinase OS=Echis carinatus sochureki OX=124223 PE=2 SV=1 | Snake venom metalloproteinases |
| 103 | 127 | tr\|E9JGE8\|E9JGE8_ECHCO | 177.04 | 15 | 15 | 1.19E+07 | 8 | 1 | 8 | Carbamidomethylation | 52979 | Metalloproteinase OS=Echis coloratus OX=64175 PE=2 SV=1 | Snake venom metalloproteinases |
| 103 | 105 | tr\|E9JGE4\|E9JGE4_ECHCO | 177.04 | 15 | 15 | 1.19E+07 | 8 | 1 | 8 | Carbamidomethylation | 52998 | Metalloproteinase OS=Echis coloratus OX=64175 PE=2 SV=1 | Snake venom metalloproteinases |
| 103 | 104 | tr\|E9JGE2\|E9JGE2_ECHCO | 177.04 | 15 | 15 | 1.19E+07 | 8 | 1 | 8 | Carbamidomethylation | 52948 | Metalloproteinase OS=Echis coloratus OX=64175 PE=2 SV=1 | Snake venom metalloproteinases |
| 103 | 102 | tr\|E9JGE3\|E9JGE3_ECHCO | 177.04 | 15 | 15 | 1.19E+07 | 8 | 1 | 8 | Carbamidomethylation | 52953 | Metalloproteinase OS=Echis coloratus OX=64175 PE=2 SV=1 | Snake venom metalloproteinases |
| 103 | 130 | tr\|E9JGE5\|E9JGE5_ECHCO | 177.04 | 15 | 15 | 1.19E+07 | 8 | 1 | 8 | Carbamidomethylation | 53565 | Metalloproteinase OS=Echis coloratus OX=64175 PE=2 SV=1 | Snake venom metalloproteinases |
| 103 | 131 | tr\|E9JGG5\|E9JGG5_ECHCO | 177.04 | 15 | 15 | 1.19E+07 | 8 | 1 | 8 | Carbamidomethylation | 53655 | Metalloproteinase OS=Echis coloratus OX=64175 PE=2 SV=1 | Snake venom metalloproteinases |
| 219 | 811 | P82463\|3SUC2_NAJKA | 67.27 | 18 | 18 | 2.46E+05 | 1 | 1 | 1 | Carbamidomethylation | 7298 | Muscarinic toxin-like protein 2 OS=Naja kaouthia OX=8649 PE=1 SV=1 | 3-Finger toxins |
| 215 | 2046 | tr\|A0A2D4LL21\|A0A2D4LL21_9SAUR | 45.19 | 9 | 9 | 4.43E+05 | 1 | 1 | 1 | Carbamidomethylation | 14536 | Pept_C1 domain-containing protein (Fragment) OS=Micrurus spixii OX=129469 PE=3 SV=1 | Snake venom serine proteinases |
| 206 | 1980 | tr\|A0A670JU78\|A0A670JU78_PODMU | 50.47 | 6 | 6 | 3.14E+06 | 1 | 1 | 1 |  | 17669 | Peptidase S1 domain-containing protein OS=Podarcis muralis OX=64176 PE=3 SV=1 | Snake venom serine proteinases |
| 206 | 1981 | tr\|A0A670K1A5\|A0A670K1A5_PODMU | 50.47 | 5 | 5 | 3.14E+06 | 1 | 1 | 1 |  | 20516 | Peptidase S1 domain-containing protein OS=Podarcis muralis OX=64176 PE=3 SV=1 | Snake venom serine proteinases |
| 206 | 1983 | tr\|A0A670JW80\|A0A670JW80_PODMU | 50.47 | 4 | 4 | 3.14E+06 | 1 | 1 | 1 |  | 25530 | Peptidase S1 domain-containing protein OS=Podarcis muralis OX=64176 PE=3 SV=1 | Snake venom serine proteinases |
| 206 | 1985 | tr\|A0A670JXY5\|A0A670JXY5_PODMU | 50.47 | 4 | 4 | 3.14E+06 | 1 | 1 | 1 |  | 28209 | Peptidase S1 domain-containing protein OS=Podarcis muralis OX=64176 PE=3 SV=1 | Snake venom serine proteinases |
| 206 | 1993 | tr\|A0A670JZC0\|A0A670JZC0_PODMU | 50.47 | 3 | 3 | 3.14E+06 | 1 | 1 | 1 |  | 39190 | Peptidase S1 domain-containing protein OS=Podarcis muralis OX=64176 PE=3 SV=1 | Snake venom serine proteinases |
| 206 | 1982 | tr\|A0A670YWE2\|A0A670YWE2_PSETE | 50.47 | 4 | 4 | 3.14E+06 | 1 | 1 | 1 |  | 25367 | Peptidase S1 domain-containing protein OS=Pseudonaja textilis OX=8673 PE=3 SV=1 | Snake venom serine proteinases |
| 213 | 1671 | tr\|A0A1W7REW7\|A0A1W7REW7_AGKCO | 48.72 | 4 | 4 | 6.73E+05 | 1 | 1 | 1 |  | 29516 | Peroxiredoxin 4 OS=Agkistrodon contortrix contortrix OX=8713 PE=4 SV=1 | Cellular components |
| 213 | 1593 | tr\|V8PB09\|V8PB09_OPHHA | 48.72 | 3 | 3 | 6.73E+05 | 1 | 1 | 1 |  | 34490 | Peroxiredoxin-4 (Fragment) OS=Ophiophagus hannah OX=8665 GN=Prdx4 PE=4 SV=1 | Cellular components |
| 213 | 2025 | P0CV91\|PRDX4_CROAT | 48.72 | 31 | 31 | 6.73E+05 | 1 | 1 | 1 |  | 4141 | Peroxiredoxin-4 (Fragments) OS=Crotalus atrox OX=8730 PE=1 SV=1 | Cellular components |
| 213 | 1592 | tr\|A0A0B8RQR7\|A0A0B8RQR7_BOIIR | 48.72 | 4 | 4 | 6.73E+05 | 1 | 1 | 1 |  | 31867 | Peroxiredoxin-4 OS=Boiga irregularis OX=92519 PE=4 SV=1 | Cellular components |
| 210 | 1619 | tr\|A0A1W7RIG1\|A0A1W7RIG1_AGKCO | 46.55 | 1 | 1 | 6.84E+05 | 1 | 1 | 1 |  | 65317 | Phosphatidylinositol binding clathrin assembly protein OS=Agkistrodon contortrix contortrix OX=8713 PE=3 SV=1 | Cellular components |
| 210 | 1610 | tr\|V8NI58\|V8NI58_OPHHA | 46.55 | 2 | 2 | 6.84E+05 | 1 | 1 | 1 |  | 51204 | Phosphatidylinositol-binding clathrin assembly protein (Fragment) OS=Ophiophagus hannah OX=8665 GN=Picalm PE=3 SV=1 | Cellular components |
| 210 | 1792 | tr\|U3F8U8\|U3F8U8_MICFL | 46.55 | 1 | 1 | 6.84E+05 | 1 | 1 | 1 |  | 71200 | Phosphatidylinositol-binding clathrin assembly protein OS=Micrurus fulvius OX=8637 PE=2 SV=1 | Cellular components |
| 210 | 1791 | tr\|A0A0B8RU30\|A0A0B8RU30_BOIIR | 46.55 | 1 | 1 | 6.84E+05 | 1 | 1 | 1 |  | 66155 | Phosphatidylinositol-binding clathrin assembly protein-like OS=Boiga irregularis OX=92519 PE=3 SV=1 | Cellular components |
| 170 | 89 | tr\|A0A141DWM1\|A0A141DWM1_9SAUR | 116.84 | 5 | 5 | 1.59E+07 | 3 | 1 | 3 | Carbamidomethylation | 91337 | Phosphodiesterase (Fragment) OS=Pseudagkistrodon rudis OX=2759634 PE=3 SV=1 | Phosphodiesterases |
| 55 | 284 | tr\|D8MIA0\|D8MIA0_BITRH | 160.99 | 24 | 24 | 3.10E+08 | 6 | 1 | 12 | Carbamidomethylation; Oxidation (M) | 28851 | Rhinocerase 2 protein (Fragment) OS=Bitis rhinoceros OX=715877 GN=rhinocerase 2 PE=3 SV=1 | Snake venom serine proteinases |
| 55 | 302 | tr\|D8MIA1\|D8MIA1_BITRH | 160.99 | 24 | 24 | 3.10E+08 | 6 | 1 | 12 | Carbamidomethylation; Oxidation (M) | 28838 | Rhinocerase 3 protein (Fragment) OS=Bitis rhinoceros OX=715877 GN=rhinocerase 3 PE=3 SV=1 | Snake venom serine proteinases |
| 78 | 457 | tr\|A0A2I7YS89\|A0A2I7YS89_9SAUR | 122.12 | 18 | 18 | 3.98E+06 | 4 | 1 | 10 | Carbamidomethylation | 27743 | Serine endopeptidase OS=Crotalus scutulatus OX=8737 PE=2 SV=1 | Snake venom serine proteinases |
| 142 | 1225 | tr\|A0A6G5ZW78\|A0A6G5ZW78_VIPAN | 116.12 | 5 | 5 | 3.53E+07 | 3 | 1 | 6 | Oxidation (M) | 28103 | Serine protease 2 OS=Vipera anatolica senliki OX=2604287 PE=2 SV=1 | Snake venom serine proteinases |
| 167 | 760 | tr\|A0A194APX9\|A0A194APX9_SISMB | 116.64 | 15 | 15 | 2.68E+06 | 4 | 1 | 5 | Carbamidomethylation | 28254 | Serine proteinase 9a OS=Sistrurus miliarius barbouri OX=8759 PE=3 SV=1 | Snake venom serine proteinases |
| 167 | 762 | tr\|A0A194ASS1\|A0A194ASS1_SISMB | 116.64 | 15 | 15 | 2.68E+06 | 4 | 1 | 5 | Carbamidomethylation | 28284 | Serine proteinase 9b OS=Sistrurus miliarius barbouri OX=8759 PE=3 SV=1 | Snake venom serine proteinases |
| 167 | 761 | tr\|A0A194AS00\|A0A194AS00_SISMB | 116.64 | 15 | 15 | 2.68E+06 | 4 | 1 | 5 | Carbamidomethylation | 28240 | Serine proteinase 9c OS=Sistrurus miliarius barbouri OX=8759 PE=3 SV=1 | Snake venom serine proteinases |
| 142 | 1456 | tr\|A0A6B7FQF8\|A0A6B7FQF8_VIPAA | 116.12 | 5 | 5 | 3.53E+07 | 3 | 1 | 6 | Oxidation (M) | 28317 | Serine proteinase SP-6 OS=Vipera ammodytes ammodytes OX=8705 PE=2 SV=1 | Snake venom serine proteinases |
| 104 | 839 | Q6T7B6\|SL2_BITGA | 149.96 | 21 | 21 | 1.79E+08 | 3 | 1 | 7 | Carbamidomethylation | 18157 | Snaclec 2 OS=Bitis gabonica OX=8694 PE=1 SV=1 | C-lectin types |
| 141 | 877 | Q6T7B5\|SL3_BITGA | 123.98 | 13 | 13 | 2.55E+08 | 2 | 1 | 5 | Carbamidomethylation; Oxidation (M) | 18095 | Snaclec 3 OS=Bitis gabonica OX=8694 PE=2 SV=1 | C-lectin types |
| 177 | 1218 | Q8JIW1\|SLUB_DEIAC | 85.68 | 6 | 6 | 1.41E+07 | 1 | 1 | 1 | Carbamidomethylation | 16726 | Snaclec agkisacutacin subunit B OS=Deinagkistrodon acutus OX=36307 PE=1 SV=2 | C-lectin types |
| 176 | 967 | tr\|A0A1W7RJY5\|A0A1W7RJY5_AGKCO | 85.8 | 4 | 4 | 8.74E+06 | 2 | 1 | 2 | Carbamidomethylation | 68968 | Snake venom metalloproteinase OS=Agkistrodon contortrix contortrix OX=8713 PE=4 SV=1 | Snake venom metalloproteinases |
| 176 | 970 | tr\|A0A194APF1\|A0A194APF1_9SAUR | 85.8 | 4 | 4 | 8.74E+06 | 2 | 1 | 2 | Carbamidomethylation | 68967 | Snake venom metalloproteinase OS=Agkistrodon piscivorus OX=8715 PE=4 SV=1 | Snake venom metalloproteinases |
| 23 | 549 | tr\|D8MIA2\|D8MIA2_BITRH | 172.3 | 17 | 17 | 4.48E+07 | 8 | 1 | 16 | Carbamidomethylation; Oxidation (M) | 28203 | Snake venom serine protease (Fragment) OS=Bitis rhinoceros OX=715877 GN=rhinocerase 4 PE=3 SV=1 | Snake venom serine proteinases |
| 13 | 165 | tr\|D5KRY0\|D5KRY0_ECHOC | 187.5 | 24 | 24 | 5.94E+07 | 10 | 1 | 20 | Carbamidomethylation; Oxidation (M) | 28236 | Snake venom serine protease (Fragment) OS=Echis ocellatus OX=99586 PE=2 SV=1 | Snake venom serine proteinases |
| 178 | 745 | Q71QJ3\|VSP01_TRIST | 100.09 | 13 | 13 | 3.60E+07 | 3 | 1 | 3 | Carbamidomethylation | 28133 | Snake venom serine protease KN1 OS=Trimeresurus stejnegeri OX=39682 PE=2 SV=1 | Snake venom serine proteinases |
| 178 | 676 | Q71QI8\|VSP10_TRIST | 100.09 | 13 | 13 | 3.60E+07 | 3 | 1 | 3 | Carbamidomethylation | 28141 | Snake venom serine protease KN10 OS=Trimeresurus stejnegeri OX=39682 PE=2 SV=1 | Snake venom serine proteinases |
| 178 | 675 | Q71QJ1\|VSP09_TRIST | 100.09 | 13 | 13 | 3.60E+07 | 3 | 1 | 3 | Carbamidomethylation | 28168 | Snake venom serine protease KN9 OS=Trimeresurus stejnegeri OX=39682 PE=2 SV=1 | Snake venom serine proteinases |
| 142 | 1457 | E5AJX2\|VSP_VIPBN | 116.12 | 5 | 5 | 3.53E+07 | 3 | 1 | 6 | Oxidation (M) | 28216 | Snake venom serine protease nikobin OS=Vipera berus nikolskii OX=1808362 GN=sp-VN PE=2 SV=1 | Snake venom serine proteinases |
| 67 | 721 | P86497\|VSPR_BITRH | 137.37 | 30 | 30 | 2.00E+08 | 5 | 1 | 10 | Carbamidomethylation; Oxidation (M) | 9978 | Snake venom serine protease rhinocerase (Fragments) OS=Bitis rhinoceros OX=715877 PE=1 SV=1 | Snake venom serine proteinases |
| 213 | 1672 | tr\|A0A2D4Q6R4\|A0A2D4Q6R4_MICSU | 48.72 | 4 | 4 | 6.73E+05 | 1 | 1 | 1 |  | 31223 | Thioredoxin domain-containing protein OS=Micrurus surinamensis OX=129470 PE=4 SV=1 | Cellular components |
| 50 | 821 | Q8AY81\|VSPST_TRIST | 141.86 | 15 | 15 | 8.62E+07 | 4 | 1 | 10 | Carbamidomethylation | 29328 | Thrombin-like enzyme stejnobin OS=Trimeresurus stejnegeri OX=39682 PE=1 SV=1 | Snake venom serine proteinases |
| 208 | 1705 | tr\|A0A2D4EJJ8\|A0A2D4EJJ8_MICCO | 64.46 | 9 | 9 | 1.17E+06 | 1 | 1 | 1 |  | 15013 | Uncharacterized protein (Fragment) OS=Micrurus corallinus OX=54390 PE=3 SV=1 | Protein family not assigned |
| 208 | 1924 | tr\|A0A2D4EJK4\|A0A2D4EJK4_MICCO | 64.46 | 10 | 10 | 1.17E+06 | 1 | 1 | 1 |  | 13014 | Uncharacterized protein (Fragment) OS=Micrurus corallinus OX=54390 PE=3 SV=1 | Protein family not assigned |
| 208 | 1925 | tr\|A0A2D4EJL2\|A0A2D4EJL2_MICCO | 64.46 | 9 | 9 | 1.17E+06 | 1 | 1 | 1 |  | 14489 | Uncharacterized protein (Fragment) OS=Micrurus corallinus OX=54390 PE=3 SV=1 | Protein family not assigned |
| 208 | 1926 | tr\|A0A2D4EJI6\|A0A2D4EJI6_MICCO | 64.46 | 8 | 8 | 1.17E+06 | 1 | 1 | 1 |  | 16323 | Uncharacterized protein (Fragment) OS=Micrurus corallinus OX=54390 PE=3 SV=1 | Protein family not assigned |
| 208 | 1706 | tr\|A0A2D4LP71\|A0A2D4LP71_9SAUR | 64.46 | 5 | 5 | 1.17E+06 | 1 | 1 | 1 |  | 24811 | Uncharacterized protein (Fragment) OS=Micrurus spixii OX=129469 PE=3 SV=1 | Protein family not assigned |
| 210 | 1786 | tr\|A0A2D4NQ92\|A0A2D4NQ92_MICSU | 46.55 | 3 | 3 | 6.84E+05 | 1 | 1 | 1 |  | 36374 | Uncharacterized protein (Fragment) OS=Micrurus surinamensis OX=129470 PE=4 SV=1 | Protein family not assigned |
| 210 | 1787 | tr\|A0A2D4NP39\|A0A2D4NP39_MICSU | 46.55 | 2 | 2 | 6.84E+05 | 1 | 1 | 1 |  | 41573 | Uncharacterized protein (Fragment) OS=Micrurus surinamensis OX=129470 PE=4 SV=1 | Protein family not assigned |
| 55 | 285 | Q6T6S7\|VSP1_BITGA | 160.99 | 23 | 23 | 3.10E+08 | 6 | 1 | 12 | Carbamidomethylation; Oxidation (M) | 28982 | Venom serine proteinase-like protein 1 OS=Bitis gabonica OX=8694 PE=1 SV=1 | Snake venom serine proteinases |
| 147 | 866 | Q6T271\|VM2BI_BITGA | 144.78 | 6 | 6 | 3.61E+06 | 4 | 1 | 4 | Carbamidomethylation | 36670 | Zinc metalloproteinase/disintegrin (Fragment) OS=Bitis gabonica OX=8694 PE=1 SV=1 | Disintegrins |
| 98 | 286 | P0C6E4\|VM2JN_PROJR | 165.98 | 14 | 14 | 1.09E+06 | 7 | 1 | 8 | Carbamidomethylation | 54880 | Zinc metalloproteinase/disintegrin OS=Protobothrops jerdonii OX=242841 PE=1 SV=1 | Snake venom metalloproteinases |
| 19 | 35 | Q4VM08\|VM3VA_MACLB | 204.52 | 20 | 20 | 1.22E+07 | 14 | 1 | 19 | Carbamidomethylation | 68710 | Zinc metalloproteinase-disintegrin-like VLAIP-A OS=Macrovipera lebetina OX=8709 PE=1 SV=1 | Disintegrins |
