# Supplementary material for: Inhibition of bacterial biofilms by the snake venom proteome
Source: Biotechnol Rep (Amst). 2023 Aug 1;39:e00810. doi: 10.1016/j.btre.2023.e00810 (PMC10407894; doi:10.1016/j.btre.2023.e00810)
Supplement: Supplementary file 3 [file mmc3.docx]

Table S3. Relative protein percentage in *Naja samarensis* venom.

| **Protein Family** | **Count^a^** | **%^b^** |
| --- | --- | --- |
| Cellular components | 77 | 35.48 |
| 3-finger toxins | 33 | 15.21 |
| Snake venom serine proteinases | 30 | 13.82 |
| Protein family not assigned | 17 | 7.83 |
| Snake venom metalloproteinases | 16 | 7.37 |
| Phospholipases A2 | 6 | 2.76 |
| Venom complement C3-likes | 6 | 2.76 |
| Cysteine-rich venom proteins | 5 | 2.30 |
| Hyaluronidases | 4 | 1.84 |
| Venom endothelial growth factors | 4 | 1.84 |
| 5’-nucleotidase family | 3 | 1.38 |
| Cathepsins | 3 | 1.38 |
| Phospholipase inhibitors | 3 | 1.38 |
| Aminopeptidases | 2 | 0.92 |
| Phospholipases B | 2 | 0.92 |
| Cystatins | 1 | 0.46 |
| L-amino-acid oxidases | 1 | 0.46 |
| Nerve growth factors | 1 | 0.46 |
| Phosphodiesterases | 1 | 0.46 |
| Venom Kunitz-type family | 1 | 0.46 |
| Vespryns | 1 | 0.46 |
| All families | 217 | 100 |

^a^ Number of proteins identified as belonging to each protein family (based on data presented in Table S1)

^b^ Percentage of each protein family relative to total number (217) of detected proteins ([number of proteins in a protein family / total number of proteins detected using LC-MSMS] x 100)
