# Supplementary material for: Inhibition of bacterial biofilms by the snake venom proteome
Source: Biotechnol Rep (Amst). 2023 Aug 1;39:e00810. doi: 10.1016/j.btre.2023.e00810 (PMC10407894; doi:10.1016/j.btre.2023.e00810)
Supplement: Supplementary file 4 [file mmc4.docx]

Table S4. Relative protein percentage in *Bitis arietans* venom.

| **Protein Family** | **Count^a^** | **%^b^** |
| --- | --- | --- |
| Snake venom serine proteinases | 35 | 21.21 |
| Cellular components | 28 | 16.97 |
| Snake venom metalloproteinases | 24 | 14.55 |
| C-lectin types | 21 | 12.73 |
| Cysteine-rich venom proteins | 10 | 6.06 |
| Protein family not assigned | 8 | 4.85 |
| Phospholipases A2 | 7 | 4.24 |
| 5’-nucleotidase family | 6 | 3.64 |
| Disintegrins | 6 | 3.64 |
| Venom Kunitz-type family | 5 | 3.03 |
| 3-Finger toxins | 3 | 1.82 |
| Aminopeptidases | 3 | 1.82 |
| Phosphodiesterases | 3 | 1.82 |
| Hyaluronidases | 2 | 1.21 |
| Cystatins | 1 | 0.61 |
| Nerve growth factors | 1 | 0.61 |
| Phospholipases B | 1 | 0.61 |
| Venom endothelial growth factors | 1 | 0.61 |
| All classes | 165 | 100 |

^a^ Number of proteins identified as belonging to each class (based on data presented in Table S2).

^b^ Percentage of each protein family relative to total number (165) of detected proteins ([number of proteins in a protein family / total number of proteins detected using LC-MSMS] x 100).
